# Risk factors for harmful alcohol consumption: a multilevel analysis*

**DOI:** 10.1590/1518-8345.7763.4686

**Published:** 2025-10-27

**Authors:** Jéssica Lima de Oliveira Pinheiro Gaia, Ana Vitória Corrêa Lima, Sheila Ramos de Oliveira, Jaqueline Lemos de Oliveira, Caroline Figueira Pereira, Divane de Vargas

**Affiliations:** 1 Escola de Enfermagem da Universidade de São Paulo, Departamento Materno-Infantil e Psiquiátrica, São Paulo, SP, Brazil; 2 Scholarship holder at the Coordenação de Aperfeiçoamento de Pessoal de Nível Superior (CAPES), Brazil; 3 Scholarship holder at the Ministério da Saúde, Instituto de Psiquiatria da USP, Brazil

**Keywords:** Health, Prevalence, Mental Health, Primary Health Care, Alcohol-Induced Disorders, Drug Users

## Abstract

to identify the prevalence of alcohol consumption patterns among Primary Health Care users and the association between risky, harmful, and probable dependence consumption patterns with sociodemographic, clinical, and behavioral characteristics.

cross-sectional study conducted with a sample of 2,178 participants who responded to the Alcohol Use Disorders Identification Test in primary health care services in the city of São Paulo. Descriptive and multiple inferential statistical analysis was performed using a hierarchical Poisson regression model with a significance level of 5%.

of the total number of participants, 18.9% met the criteria for harmful alcohol consumption. Determinants for risky and harmful alcohol consumption were male gender (PR = 1.89; 95% CI 1.57–2.28; p < 0.001), black race/color (PR = 1.65; 95% CI 1.30 - 2.10; p < 0.001), and income ≥ 10 minimum wages (PR = 1.78; 95% CI 1.07 - 2.99; p = 0.028). For probable dependence, lower educational level (PR = 13.70; 95% CI 1.56–117.63; p = 0.017) and reporting a diagnosis of depression (PR = 2.72; 95% CI 1.37–5.41; p = 0.005) were determinants.

the prevalence of alcohol use disorders among users of primary health care services in São Paulo was higher than that observed in previous studies, suggesting that this segment of the population seeks health care at these services, highlighting the potential for screening and early diagnosis of the population.

## Destacados

**(1)** High prevalence of alcohol use disorders in primary health care. **(2)** Risk factors: gender, race/color, income, low education, and depression. **(3)** Need for screening and intervention strategies.

## Introduction

Harmful alcohol consumption is associated with harm to oneself and others, such as family members, friends, coworkers, or strangers. This is because it directly increases the risk of developing health problems, such as cardiovascular and liver diseases, as well as engaging in risky behaviors that can result in violence and traffic accidents^(1)^. In addition, alcohol negatively impacts individuals’ mental health and quality of life, a fact that has been observed even more intensely during the COVID-19 pandemic^(2)^.

Brazil is among the countries in the Americas with the highest per capita alcohol consumption rates. According to information from the Public Health Observatory, 35.4% of the Brazilian population drinks alcohol, with a higher prevalence among adults living in Brazilian capitals, who consume the substance at least once a week, as revealed by data from the Surveillance of Risk and Protective Factors for Chronic Diseases by Telephone Survey (Vigitel)^(3)^.

Primary Health Care (PHC) is an important space for screening and identifying harmful alcohol consumption, as it is the gateway for the population to health care, covering 83% of the population, considering the national average^(4)^. Furthermore, there is evidence that about 20% of people receiving treatment for morbidities in PHC report harmful alcohol consumption^(5-6)^. Among the most common clinical conditions in PHC, frequently associated with harmful alcohol consumption, are hypertension, insomnia, liver problems, depression, and anxiety^(7)^.

In this context, PHC shows great potential for screening and early diagnosis of users who engage in harmful alcohol consumption, due to its ease of access, ability to establish links, and comprehensive approach, which includes health promotion and disease prevention actions, consolidating itself as an essential care device^(6)^.

Despite this potential, there are barriers that hinder, and even prevent, the effective search for these patients in these settings. In general, the challenges are mainly marked by organizational difficulties in institutions, in relation to high demands for care, the burden of the disorder, associated with the scarcity of properly implemented interventions in PHC, the stigma and prejudice faced, and the low prevalence of users who have their alcohol consumption assessed by health professionals^(8-9)^. These factors justify the implementation of screening strategies in PHC for the early identification of risk for harmful alcohol consumption and effective interventions for those who abuse the substance. It is understood that such patterns are a risk factor for the health of the population, especially those with other associated chronic diseases.

In this sense, considering that there is little Brazilian research that has sought to identify the alcohol consumption patterns of individuals seeking health care in PHC services, this study aims to identify the prevalence of alcohol consumption patterns among PHC users, and the association between patterns of risky, harmful, and probable dependence consumption with sociodemographic, clinical, and behavioral characteristics.

## Method

### Study design and participants

A cross-sectional study, conducted between November 2021 and May 2023 with users over the age of 18 treated at Primary Care Center (PCCs) in the municipality of São Paulo. Data collection was carried out in 13 of the 479 PCCs in the municipality of São Paulo. The selected units are distributed across different regions of the city, including: one in the North, two in the South, two in the Southeast, two in the East, two in the West, and four in the Center. Despite the convenience sample, the selection sought to consider geographic and sociodemographic diversity, with representatives from each region of the municipality. Potential participants were recruited using the unit’s daily appointment schedule. The exclusion criteria were: presenting a low-risk/abstinence pattern, being in treatment for alcohol-related problems, being a person with hearing impairment, and presenting noticeable signs of mental confusion or intoxication by psychoactive substances during data collection. To assess signs of mental confusion, questions were asked about the day of the week, month, and year, as well as identifying the time of the interview (morning, afternoon, or evening), according to the time it was conducted. To assess signs of intoxication by psychoactive substances, an evaluation of the user’s speech was added to determine if it was slurred, and the coherence of their responses.

### Data collection instruments

The Alcohol Use Disorders Identification Test (AUDIT) instrument is used exclusively to identify problems associated with alcohol consumption. It has been validated for the Brazilian population in a study of patients treated in the context of primary care, identifying it as a useful tool for screening alcohol consumption patterns in this population, with high validity, reliability (Cronbach’s alpha > 0.80), and accuracy (sensitivity > 0.80; specificity > 0.70) ^(10 )^ . It is an instrument composed of 10 questions, each of which varies on a scale between 0 and 4 points, resulting in a total score between 0 and 40 points at the end of the questionnaire ^(11 )^ . According to the score obtained in the instrument, four classifications can be assigned to the pattern of alcohol consumption: Zone I: low-risk consumption or abstainers (between 0 and 7 points); Zone II: risky consumption (between 8 and 15 points); Zone III: harmful consumption (between 16 and 19 points); and Zone IV: probable dependence (between 20 and 40 points). 

In addition to the AUDIT, a questionnaire was administered with questions related to sociodemographic characteristics: age, race/color, gender, marital status, education, occupation, family income, lifestyle, physical activity, comorbidities, clinical and psychiatric history. Questions related to COVID-19 were also investigated, such as whether the participant had been infected during the pandemic, whether they had been hospitalized or admitted to an intensive care unit (ICU), whether they had lost family members as a result of the disease, and whether they had noticed a change in their alcohol consumption pattern as a result of the pandemic.

It is understood that questions related to COVID-19 are justified, since previous studies point out that there was an increase in alcohol consumption during the pandemic as a way of coping with both social and emotional issues due to hospitalizations, loss of family members, and difficulties in mourning due to the absence of adequate funeral rituals and prolonged confinement ^(12-13 )^ . 

### Data collection

The research team had access to the contact details (telephone numbers) of potential participants in the service schedules. The managers of the health units sent the schedules monthly to the research group’s email address, from which the telephone contacts were distributed among the research collectors, who made five attempts, at different times, to contact users over the age of 18, inviting them to participate in the study. For those who agreed to participate, the reason for the research and its objectives were explained during the recorded call, and the Informed Consent Form (ICF) was read aloud, with a copy of the form made available to the participant via WhatsApp.

### Data analysis

The explanatory variables in this study were organized hierarchically from the most proximal (innate to the individual) to the most distal (contextual) in three distinct hierarchical levels. Level 1: gender (male and female), age (continuous years), race/color (black; brown; white; Asian; indigenous; or undeclared). Level 2 (demographic): marital status (single; married; cohabiting/living together; divorced/separated; widowed), education (illiterate; incomplete elementary school; complete elementary school; incomplete high school; complete high school; incomplete higher education; complete higher education; incomplete postgraduate education; complete postgraduate education; unknown), family income (< 1 minimum wage; 1 to 5 minimum wages; 6 to 10 minimum wages; > 10 minimum wages; unknown). Level 3 (health): engages in physical activity (yes; no), high blood pressure (yes; no); diabetes (yes; no); cholesterol (yes; no); gastric problems (yes; no); depression (yes; no).

The variables were treated descriptively and inferentially using IBM SPSS Statistics software. Categorical variables were described using absolute (n) and relative (%) frequencies, while the only continuous variable was described using mean and standard deviation. Inferential analysis was performed using robust Poisson regression to calculate prevalence ratios (PR), p-values, and 95% confidence intervals (95% CI).

The hierarchical model was used as the basis for multivariate statistical analyses. At the first level, all variables were included simultaneously and adjusted to each other. From the second level onwards, the model included the variables from the previous level that had a p-value < 0.20 in the bivariate analysis, in addition to the new variables from the level in question, adjusted to each other and in relation to the variables retained from the previous levels. This procedure was repeated for the third level. We chose to exclude the least significant variables from each level in order to develop final models that were more parsimonious, less complex, and better adjusted. This approach offers advantages such as control of potential confounding variables and reduction of the risk of inappropriate adjustments for variables that may act as mediators in the relationship between the exposure variables and the outcome ^(14 )^ . 

To test the linearity of the age variable, it underwent a power transformation of 0.5. Models with the original variable were compared with models in which the transformed variable was included. Models without the transformation showed a better fit, with lower corrected Akaike Information Criterion and deviance. Therefore, age was maintained as a continuous variable without transformation in the adopted models.

### Ethical procedures

The study was approved by the Research Ethics Committee, under opinion No. 4,342,492. All individuals participated after agreeing to the informed consent form.

## Results

### Sample characteristics

**Figure 1-:**
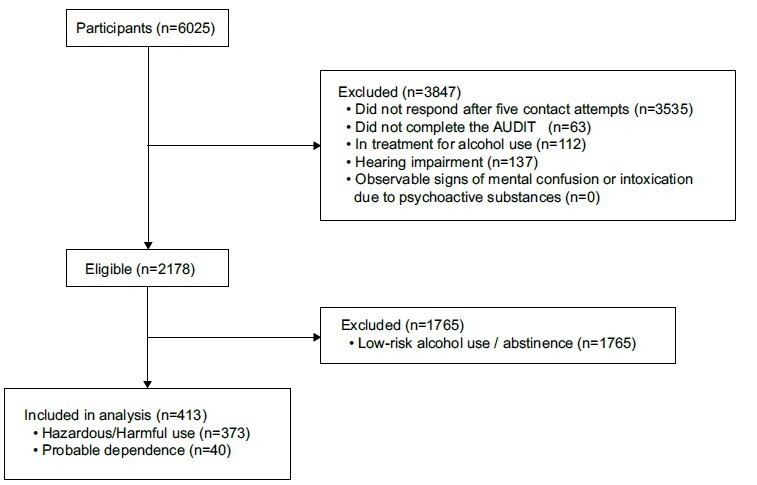
Participant eligibility flowchart. São Paulo, SP, Brazil, 2023

Of the sample, 69.8% were female, with an average age of 44.9 years, and 42.2% declared their race/color to be white. Regarding marital status, 36.5% declared themselves to be single, while regarding education, 36.3% declared having completed high school. The majority of the sample declared having a family income between 1 and 5 minimum wages (64.8%). Regarding health characteristics, 48.0% declared performing some physical activity, 31.0% reported high blood pressure, 15.1% diabetes, 19.2% cholesterol, 27.0% gastric problems, and 17.6% depression. Regarding contextual variables related to the COVID-19 pandemic, 33.6% reported having been diagnosed with the disease, 1.9% required hospitalization for COVID-19, and 0.7% required ICU admission due to complications from the disease, while 42.4% of the subjects reported having lost someone to COVID-19.

Regarding alcohol consumption patterns in the sample, 17.1% presented risky and harmful consumption (AUDIT from 8 to 19 points), while 1.8% of the sample was characterized as having possible dependence on alcohol consumption (AUDIT ≥ 20 points). Of the total sample, 18.9% met the criteria for harmful alcohol consumption, as shown in Table 1.

**Table 1-:** Prevalence of alcohol use risk groups in Primary Health Care users stratified by sex (n = 373). São Paulo, SP, Brazil, 2023

**Sex**	**Risky/harmful consumption**			**Probable dependence**		
	n*	**Prevalence**	**95%CI** ^†^	n*	**Prevalence**	**95%CI** ^†^
Male	156	7.2%	6.1; 8.2%	16	0.7%	0.4; 1.1%
Female	217	9.9%	8.7; 11.2%	24	1.1%	0.7; 1.5%
Total	373	17.1%	15.7; 18.3%	40	1.8%	1.3; 2.4%

*n = Sample; ^†^ CI = Confidence Interval

A hierarchical multivariate analysis was performed for characteristics associated with risky and harmful alcohol consumption. This analysis identified that risky/harmful alcohol consumption was more prevalent among males than females (PR = 1.89; 95% CI 1.57–2.28; p < 0.001), among people who identified themselves as black compared to white (PR = 1.65; 95% CI 1.30 - 2.10; p < 0.001), among people with an income ≥ 10 minimum wages when compared to those receiving <1 minimum wage (PR = 1.78; 95% CI 1.07–2.99; p = 0.028). In addition, it is evident that the outcome becomes less prevalent with advancing age (PR = 0.98; 95% CI 0.97–0.99; p < 0.001), as shown in Table 2 . 

**Table 2-:** Hierarchical multivariate analysis for characteristics associated with risky and harmful alcohol consumption in primary health care users (n = 373). São Paulo, SP, Brazil, 2023

	**Hierarchical analysis**								
	**Model 1**			**Model 2**			**Model 3**		
**Level 1**	PR*	p-value ^†^	95% CI ^‡^	PR*	p-value ^†^	95%CI ^‡^	PR*	p-value ^†^	95%CI ^‡^
Sex	**p <0.001** ^§^			**p <0.001** ^§^			**p <0.001** ^§^		
Male	1.84	<0.001	1.53; 2.21	1.82	<0.001	1.51; 2.19	1.89	<0.001	1.57; 2.28
Female	1			1			1		
Age	**<0.001**			**<0.001**			**<0.001**		
Continuous years	0.98	<0.001	0.97; 0.99	0.97	<0.001	0.97; 0.98	0.98	<0.001	0.97; 0.99
Race/color	**p =0.002**			**p =0.004**			**p =0.002**		
Not stated	1.47	0.239	0.77; 2.79	1.39	0.306	0.74; 2.59	1.42	0.292	0.74; 2.72
Black	1.65	<0.001	1.30; 2.10	1.62	<0.001	1.26; 2.07	1.65	<0.001	1.30; 2.10
Brown	1.09	0.423	0.88; 1.36	1.09	0.444	0.87; 1.38	1.10	0.381	0.89; 1.37
Oriental	1.18	0.600	0.64; 2.16	1.18	0.595	0.64; 2.17	1.13	0.686	0.62; 2.08
Indigenous	0.70	0.592	0.19; 2.56	0.71	0.597	0.20; 2.55	0.69	0.567	0.19; 2.47
White	1			1			1		
**Level 2**									
Marital status				p =0.244					
Widowed				1.19	0.604	0.62; 2.26			
Single				1.26	0.075	0.98; 1.67			
Cohabiting/Living together				1.23	0.174	0.91; 1.65			
Divorced/Separated				1.46	0.034	1.03; 2.07			
Married				1					
Education				p =0.763					
Completed elementary school				1.07	0.745	0.69; 1.67			
Incomplete elementary school				1.16	0.392	0.82; 1.65			
Completed high school				0.91	0.502	0.69; 1.20			
Incomplete high school				1.22	0.280	0.85; 1.77			
Incomplete higher education				0.94	0.742	0.67; 1.33			
Completed postgraduate studies				1.09	0.720	0.68; 1.76			
Incomplete postgraduate studies				0.89	0.780	0.41; 1.94			
Don’t know				0.82	0.846	0.12; 5.86			
Higher education completed				1					
Family income				p =0.189			p =0.139		
From 1 to 5 minimum wages ^||^				1.14	0.387	0.85; 1.54	1.13	0.416	0.84; 1.51
From 6 to 10 minimum wages ^||^				1.28	0.280	0.81; 2.01	1.24	0.334	0.80; 1.90
≥ 10 minimum wages ^||^				1.83	0.028	1.07; 3.16	1.78	0.028	1.07; 2.99
Don’t know				0.99	0.967	0.67; 1.47	0.53	0.751	0.64; 1.38
< 1 minimum wage ^||^				1			1		
**Level 3**	**Hierarchical analysis**								
	**Model 1**			**Model 2**			**Model 3**		
	PR*	p-value ^†^	95% CI ^‡^	PR*	p-value ^†^	95%CI ^‡^	PR*	p-value ^†^	95%CI ^‡^
Engages in physical activity							p =0.141		
No							1.15	0.141	0.95; 1.38
Yes							1		
High blood pressure							p =0.084		
No							1.28	0.084	0.97; 1.63
Yes							1		
Diabetes							p =0.393		
No							1.17	0.393	0.82; 1.67
Yes							1		
Cholesterol							p =0.075		
No							1.33	0.075	0.97; 1.82
Yes							1		
Gastric problems							p =0.378		
No							0.91	0.378	0.74; 1.12
Yes							1		
Depression							p =0.100		
Yes							1.20	0.100	0.96; 1.51
No							1		
Corrected Akaike Information Criterion	1934.451			1951.693			1934.412		

Note: Model 1 (adjusted for all Level 1 variables); Model 2 (adjusted for variables with p<0.200 in the overall effect test of the level above and for all Level 2 variables); Model 3 (adjusted for variables with p<0.200 in the overall effect test of the above levels and for all Level 3 variables; The category “illiterate” was removed from the analysis due to the low frequency of individuals with the outcome and generating problems of Hessian matrix singularity in the model. The Akaike Information Criterion (AIC) evaluates statistical models by balancing the fit to the data with the complexity of the parameters. Models with lower AIC are preferable, as they avoid overfitting without losing performance. *PR = Prevalence ratio; ^†^ p-value = Significance level; ^‡^ CI = Confidence interval; ^§^ p <0.001 = Statistical significance; ^||^ SM = Minimum wage in Brazil in 2023, R$ 1,320.00

In Table 2 , model 2 showed the worst performance (AICc = 1951.693), suggesting that the addition of demographic variables worsened the fit, probably because they did not contribute significantly to explaining the outcome. In turn, model 3 presented the lowest AICc (1934.412), indicating the best balance between fit and complexity represented by the number of variables included. 

The hierarchical multivariate analysis for characteristics associated with probable alcohol dependence (AUDIT ≥ 20 points) showed that this outcome was more prevalent among users with lower levels of education (PR = 13.70; 95% CI 1.56–117.63; p = 0.017) and those who reported incomplete higher education (PR = 4.10; 95% CI 1.09–15.42; p = 0.037) than those with complete higher education. It was also more prevalent among users with depression than those who denied the pathology (PR = 2.72; 95% CI 1.37–5.41; p = 0.005), as shown in Table 3 . 

**Table 3-:** Hierarchical multivariate analysis for characteristics associated with probable alcohol dependence in primary health care users (n = 40). São Paulo, SP, Brazil, 2023

**Level 1**	**Hierarchical analysis**								
**Model 1**			**Model 2**			**Model 3**		
	PR*	p-value ^†^	95% CI ^‡^	PR*	p-value ^†^	^‡^ 95% CI ^‡^	PR*	p-value ^†^	95% CI ^‡^
Sex	p =0.174 ^§^			p =0.153 ^§^			p =0.106 ^§^		
Male	1.58	0.174	0.82; 3.05	1.59	0.153	0.84; 3.01	1.75	0.106	0.89; 3.45
Female	1			1			1		
Age	**p =0.030**			**p =0.006**			p =0.269		
Continuous years	0.98	0.030	0.96; 1.00	0.97	0.006	0.96; 0.99	0.99	0.269	0.97; 1.01
Race/color	p =0.774								
Not declared	1.89	0.534	0.25; 13.94						
Black	0.94	0.904	0.33; 2.63						
Brown	1.34	0.416	0.66; 2.73						
Oriental	1.44	0.720	0.19; 10.63						
Indigenous	3.51	0.203	0.51; 24.34						
White	1								
**Level 2**									
Marital status	p =0.208								
Widowed				2.58	0.224	0.56; 11.89			
Single				1.43	0.434	0.58; 3.52			
Cohabiting/Living together				1.33	0.587	0.48; 3.68			
Divorced/Separated				3.31	**0.021**	1.20; 9.13			
Married				1					
Education				p =0.121			p =0.191		
Illiterate				15.19	**0.015**	1.70; 135.98	13.70	**0.017**	1.60; 117.63
Complete elementary education				3.25	0.142	0.67; 15.66	3.07	0.171	0.62; 15.29
Incomplete elementary education				4.33	**0.025**	1.21; 15.54	3.88	0.051	0.99; 15.14
Complete high school education				2.33	0.158	0.72; 7.55	2.21	0.214	0.63; 7.72
Incomplete high school education				1.64	0.574	0.29; 9.24	1.42	0.694	0.24; 8.29
Incomplete higher education				4.67	**0.020**	1.28; 17.11	4.10	**0.037**	1.09; 15.42
Complete postgraduate education				1.25	0.854	0.12; 13.48	1.26	0.842	0.13; 12.21
Complete higher education				1			1		
Family income				p =0.390					
From 1 to 5 SM ^||^				0.91	0.846	0.37; 2.24			
From 5 to 10 SM ^||^				2.09	0.234	0.62; 7.06			
≥ 10 SM ^||^				1.10	0.933	0.12; 9.70			
Don’t know				0.49	0.317	0.12; 1.99			
< 1 SM ^||^				1					
**Level 3**									
Engages in physical activity							p =0.501		
No							1.26	0.501	0.64; 2.48
Yes							1		
High blood pressure							p =0.400		
No							1.50	0.400	0.58; 3.82
Yes							1		
Diabetes							p =0.119		
No							3.11	0.119	0.75; 12.97
Yes							1		
Cholesterol							p =0.961		
No							0.98	0.961	0.43; 2.23
Yes							1		
Gastric problems							p =0.851		
No							0.94	0.851	0.47; 1.86
Yes							1		
Depression							**p =0.005**		
Yes							2.72	0.005	1.37; 5.41
No							1		
Corrected Akaike Information Criterion	399.086			406.284			397.777		

Note: Model 1 (adjusted for all Level 1 variables); Model 2 (adjusted for variables with p<0.200 in the overall effect test of the level above and for all Level 2 variables); Model 3 (adjusted for variables with p<0.200 in the overall effect test of the above levels and for all Level 3 variables); The categories “incomplete postgraduate education” and “don’t know” of the education variable were removed from the analysis due to the low frequency of individuals with the outcome and generated problems of singularity of the Hessian matrix in the model. The Akaike Information Criterion (AIC) evaluates statistical models by balancing the fit to the data with the complexity of the parameters. Models with lower AIC are preferable because they avoid overfitting without losing performance. *PR = Prevalence ratio; ^†^ p-value = Significance level; ^‡^ CI = Confidence interval; ^§^ p <0.001 = Statistical significance; ^||^ SM = Minimum wage in Brazil in 2023, R$ 1,320.00

In Table 3 , model 2 showed the worst performance (AICc = 406.284), suggesting that the addition of demographic variables worsened the fit, probably because they did not contribute significantly to explaining the outcome. In turn, model 3 presented the lowest AICc (397.77), indicating the best balance between fit and complexity represented by the number of variables included. 

## Discussion

The present study aimed to identify the prevalence of alcohol consumption patterns among PHC users and the association between risky, harmful, and probable dependence consumption patterns with sociodemographic, clinical, and behavioral characteristics. The results on the prevalence of risky/harmful consumption in the present study were 17.1%, differing from a survey in the municipality of São Paulo, that identified a prevalence of 7.7% in the general population ^(15 )^ . 

When broken down by gender, women in the present study had a prevalence of risky/harmful consumption of 9.9%, compared to 3.7% in the municipal survey; in the male population, it was 7.2% and 12.4%, respectively. It should be noted that 70% of the sample was composed of women, corroborating a previous study ^(16 )^ . In this context, this may reflect greater awareness of their own consumption, resulting from continuous monitoring at the PCC and greater awareness of their health-disease process. 

Despite this, in the multivariate analysis, males were associated with risky/harmful consumption patterns. It is understood that this pattern is closely linked to cultural factors and the attribution of distinct social roles. While alcohol consumption by men may be associated with the expression of masculinity and sociability, women often face more restrictive social norms that discourage the same behavior ^(17 )^ . 

The findings present clear evidence of risky and harmful consumption among young people, consistent with previous research that highlights a worrying association between early alcohol consumption and the adoption of other risky behaviors ^(18 )^ . It is understood that this high prevalence of alcohol use among young people is associated with easy access to the substance, coupled with social and cultural influences, contributing to the trivialization of its consumption ^(19-20 )^ . 

Risky/harmful consumption was also associated with people who self-identified as black, revealing the intersection between structural racism and mental health, in which individuals are diagnosed based on their skin color, in addition to having greater difficulties in accessing health services ^(21-23 )^ . A study conducted with veterans found that black men were 23% to 109% more likely to receive a diagnosis of alcohol use disorder than white men, highlighting the diagnostic bias to which black people are subjected ^(21 )^ . In addition, studies highlight the black population’s lower access to health care compared to the white population, emphasizing the negative consequences on the health outcomes of this population ^(22-23 )^ . 

It is understood that inequalities are perpetuated by institutional barriers and experiences of discrimination in health services, where certain social groups are systematically excluded and marginalized in alcohol and other drug care policies, reproducing historical and institutional violence ^(24-26 )^ . This scenario demands an urgent reformulation of public mental health policies and professional training, incorporating an anti-racist and culturally sensitive perspective that recognizes and actively addresses the social markers of inequality. Such an approach is fundamental to developing care strategies that promote equity and break the cycles of exclusion and stigmatization. 

Although the study found an association between risky/harmful alcohol consumption and higher income, this association is complex. Studies indicate that low-income individuals tend to consume alcohol more frequently and intensely in response to adverse socioeconomic and psychological factors ^(24,27 )^ , whereas high-income individuals consume alcohol in large quantities, driven by cultural and social factors such as events and status display ^(28-29 )^ . In addition, access to health care and support is greater among high-income individuals, which can aggravate outcomes for the economically vulnerable, who face significant barriers to seeking treatment ^(30 )^ . 

It should also be noted that the pandemic has intensified these challenges. Studies point to important implications for accessibility to consumption due to financial impacts, as well as public policies to control consumption ^(31 )^ . Although no significant association was observed between harmful alcohol consumption and the direct impacts of the pandemic in the sample analyzed, it is important to highlight that the literature points to a generalized increase in health risk behaviors during the period of social isolation ^(32 )^ . 

This scenario suggests that the health crisis context may have exacerbated risk factors for problematic alcohol use in other populations, making it essential to implement preventive measures in PHC, considering the multiple dimensions that influence consumption patterns and their relationship with the socioeconomic factors of the population.

The most self-reported comorbidities were hypertension, diabetes mellitus, gastritis, and cholesterol. Despite the lack of statistical significance, the data corroborate the literature, which points to a high prevalence of preexisting chronic diseases among individuals who misuse alcohol in PHC, resulting in the worsening of such conditions ^(33-34 )^ . The lack of a systematic approach to alcohol consumption contributes to underdiagnosis and the absence of prevention and early treatment of alcohol-related disorders ^(35 )^ , highlighting the need for greater attention to screening and interventions in PHC. 

It is important to note that, as this is a cross-sectional study, it is not possible to establish causal or temporal relationships between alcohol consumption and the identified comorbidities. The complexity of these associations may go beyond the limits of a cross-sectional study, since sociocultural, economic, and behavioral factors, which were not included in the statistical model, may play important roles in these associations. Longitudinal studies incorporating a wider range of variables are recommended to elucidate these issues, which are suggestions for future studies.

The results of this study pointed to an association between educational level, self-reported depression, and probable alcohol dependence, in line with previous studies ^(20,36 )^ . It is understood that education plays a crucial role in the development of coping skills and the formation of social support networks, factors that contribute to the prevention of dependence. On the other hand, its absence can limit access to these protective tools, favoring risky behaviors ^(37-38 )^ . Considering that alcohol is often used as a strategy to alleviate symptoms of mental distress, such as anxiety and sadness, there is a need for integrated approaches that consider both psychological and social aspects for the best referral of cases ^(22-34 )^ . 

As evidenced by the results of this study, problems related to alcohol consumption are influenced by both individual factors and social determinants. Therefore, PHC, with its comprehensive and territorialized nature, presents itself as a strategic space for health promotion, disease prevention, and harm reduction actions. However, for these strategies to be effective, it is essential to overcome existing organizational barriers, such as high demand for care and professional overload, in addition to investing in team training for a stigma-free and welcoming approach.

This study should be interpreted in the context of limitations, among which are that all results are based on self-reporting, which may lead to omission of information or underestimation of the frequency and amount of alcohol consumed, limitations of access to specialized services, due to possible social desirability biases. Strategies were implemented to standardize data collection and align researchers to minimize such bias.

In addition, the cross-sectional design also limits causal inference, since it is not possible to determine the temporality between exposure (alcohol consumption) and outcomes (such as comorbidities and mental disorders). Data collection during the pandemic may have introduced memory bias, in addition to the lack of pre-pandemic comparative data, limiting the ability to assess specific changes during this period. Furthermore, since the sample is not representative of the municipality or other regions, it is impossible to generalize the data.

Furthermore, variables such as religion/religiosity, social support, other known protective factors, and the exclusion of deaf people were not considered, which may represent an additional limitation. However, the findings of this study may support future research on the prevalence of alcohol consumption patterns among users of PHC services, which consider the aspects listed above.

## Conclusion

It is concluded that the study, when seeking to identify the prevalence of alcohol consumption patterns among PHC users in the city of São Paulo, indicated that harmful alcohol consumption is mainly associated with male users, younger, black, and with an income equal to or greater than 10 minimum wages. The search for care in PHC occurs, in the majority, for other health problems, which reinforces the importance of this level of care as a strategic scenario for the early identification of harmful alcohol consumption and the implementation of preventive measures.

## Data Availability

All data generated or analysed during this study are included in this published article.
