# Supplementary material for: Risk factors for harmful alcohol consumption: a multilevel analysis
Source: Rev Lat Am Enfermagem. 2025 Oct 27;33:e4686. doi: 10.1590/1518-8345.7763.4686 (PMC12562657; doi:10.1590/1518-8345.7763.4686)
Supplement: Translation

# Translation: Fatores de risco para o consumo prejudicial de álcool: análise multinível*

**DOI:** 10.1590/1518-8345.7763.4687

**Authors:** Jéssica Lima de Oliveira Pinheiro Gaia, Ana Vitória Corrêa Lima, Sheila Ramos de Oliveira, Jaqueline Lemos de Oliveira, Caroline Figueira Pereira, Divane de Vargas

**Affiliations:** 1 Escola de Enfermagem da Universidade de São Paulo, Departamento Materno-Infantil e Psiquiátrica, São Paulo, SP, Brasil; 2 Bolsista da Coordenação de Aperfeiçoamento de Pessoal de Nível Superior (CAPES), Brasil; 3 Bolsista do Ministério da Saúde, Instituto de Psiquiatria da USP, Brasil

**Keywords:** Saúde, Prevalência, Saúde Mental, Atenção Primária à Saúde, Transtornos Induzidos por Álcool, Usuários de Drogas

## Abstract

identificar a prevalência dos padrões de consumo de álcool entre usuários da Atenção Primária à Saúde e a associação entre os padrões de consumo de risco, nocivo e provável dependência, com características sociodemográficas, clínicas e comportamentais.

estudo transversal, realizado com uma amostra de 2178 participantes que responderam o *Alcohol Use Disorders Identification Test*, em serviços de Atenção Primária à Saúde do município de São Paulo. Realizou-se análise estatística descritiva e inferencial múltipla com modelo hierarquizado de regressão de Poisson com nível de significância de 5%.

do total de participantes, 18,9% preencheram critérios para consumo prejudicial de álcool. Foram determinantes para o consumo de risco e nocivo de álcool o sexo masculino (RP = 1,89; IC95% 1,57 - 2,28; p < 0,001), raça/cor preta (RP = 1,65; IC95% 1,30 - 2,10; p < 0,001) e renda ≥ 10 salários-mínimos (RP = 1,78; IC95% 1,07 – 2,99; p = 0,028). Para a provável dependência foram determinantes menor nível de escolaridade (RP = 13,70; IC95% 1,56 – 117,63; p = 0,017) e referir ter diagnóstico de depressão (RP = 2,72; IC95% 1,37 – 5,41; p = 0,005).

a prevalência de transtornos por consumo de álcool entre usuários dos serviços de atenção primária à saúde de São Paulo mostrou-se maior do que observada em estudos prévios, sugerindo que essa parcela da população busca atendimento de saúde nesses serviços, destacando a potencialidade na triagem e diagnóstico precoce da população.

## Destaques

**(1)** Alta prevalência de transtornos por uso de álcool na Atenção Primária à Saúde. **(2)** Fatores de risco: sexo, raça/cor, renda, baixa escolaridade e depressão. **(3)** Necessidade de estratégias de rastreio e intervenção.

## Introdução

O consumo prejudicial de álcool está associado a danos a si próprio e a terceiros, como familiares, amigos, colegas de trabalho ou estranhos. Isso ocorre porque aumenta-se diretamente o risco de desenvolver problemas de saúde, como doenças cardiovasculares e hepáticas, bem como de ter comportamentos de risco que podem resultar em violência e acidentes de trânsito^(1)^. Além disso, o álcool impacta negativamente a saúde mental e a qualidade de vida dos indivíduos, fato que foi observado de forma ainda mais intensa durante a pandemia de COVID-19^(2)^.

O Brasil está entre os países das Américas com os maiores índices de consumo de álcool *per capita*. De acordo com informações do Observatório de Saúde Pública, 35,4% da população brasileira ingerem bebidas alcoólicas, com maior prevalência entre adultos residentes nas capitais brasileiras, que consomem a substância pelo menos uma vez por semana, conforme revelam os dados da Vigilância de Fatores de Risco e Proteção para Doenças Crônicas por Inquérito Telefônico (Vigitel)^(3)^.

A Atenção Primária à Saúde (APS) se configura como um espaço importante para o rastreio e identificação do consumo prejudicial de álcool, isso porque é a porta de entrada da população para o atendimento em saúde, perfazendo uma taxa de cobertura de 83% da população, considerando a média nacional^(4)^. Ademais, existem evidências de que cerca de 20% das pessoas que recebem tratamento por morbidades na APS referem fazer consumo prejudicial de álcool^(5-6)^. Entre os quadros clínicos mais comuns na APS, frequentemente associados ao consumo prejudicial de álcool estão: hipertensão, insônia, problemas hepáticos, depressão e ansiedade^(7)^.

Nesse contexto, a APS demonstra grande potencialidade para a triagem e o diagnóstico precoce dos usuários que fazem o consumo prejudicial de álcool, devido à sua facilidade de acesso, à capacidade de estabelecer vínculos e à abordagem integral, que inclui ações de promoção da saúde e prevenção de agravos, consolidando-se como um dispositivo essencial de cuidado^(6)^.

Apesar dessas potencialidades, existem barreiras que dificultam e até impedem que a busca efetiva desses pacientes aconteça nesses cenários. Em geral, os desafios são marcados principalmente por dificuldades organizacionais nas instituições em relação a altas demandas de cuidados, o fardo do transtorno associado à escassez de intervenções devidamente implementadas na APS, o estigma e preconceito enfrentados, além da baixa prevalência de usuários que têm seu consumo de álcool avaliado pelos profissionais de saúde^(8-9)^. Tais fatores justificam a implementação de estratégias de rastreio na APS, para que seja realizada a identificação precoce do risco para o consumo prejudicial de álcool e intervenções eficientes para aqueles que fazem o consumo abusivo da substância. Entende-se que tais padrões se apresentam como fator de risco para a saúde da população, principalmente que tenham outras doenças crônicas associadas.

Nesse sentido, considerando que são escassas as pesquisas brasileiras que tenham se ocupado de identificar o padrão de consumo de álcool de indivíduos que buscam por atendimento de saúde em serviços de APS, objetiva-se, neste estudo, identificar a prevalência dos padrões de consumo de álcool entre usuários da APS e a associação entre os padrões de consumo de risco, nocivo e provável dependência, com características sociodemográficas, clínicas e comportamentais.

## Método

### Delineamento do estudo e participantes

Estudo transversal, ocorrido entre novembro de 2021 e maio de 2023, com usuários maiores de 18 anos atendidos em Unidades Básicas de Saúde (UBS) do município de São Paulo. A coleta de dados foi realizada em 13 das 479 UBS do município de São Paulo. As unidades escolhidas estão distribuídas em diferentes regiões da cidade, incluindo: uma da Norte, duas da Sul, duas da Sudeste, duas da Leste, duas da Oeste e quatro da Central. Apesar da amostra por conveniência, a seleção buscou considerar a diversidade geográfica e sociodemográfica, com representantes de cada região do município. Os possíveis participantes foram recrutados utilizando-se a agenda diária de atendimentos da unidade. Os critérios de exclusão foram: apresentar padrão de baixo risco/abstinência, estar em tratamento para problemas relacionados ao consumo do álcool, ser pessoa com deficiência auditiva e apresentar sinais perceptíveis de confusão mental ou intoxicação por substâncias psicoativas durante a coleta de dados. Para avaliar os sinais de confusão mental foram realizadas perguntas sobre o dia da semana, o mês, o ano, bem como identificar qual era o período da entrevista manhã, tarde ou noite, de acordo com o horário que era realizada. Para avaliar sinais de intoxicação por substâncias psicoativas adicionou-se a avaliação da fala do usuário se estava pastosa e a coerência das respostas.

### Instrumentos para coleta de dados

O instrumento *Alcohol Use Disorders Identification Test* (AUDIT) é utilizado exclusivamente para a identificação de problemas associados ao consumo de álcool, foi validado para a população brasileira em estudo com pacientes atendidos no contexto da APS, identificando ser um instrumento útil para o rastreio do padrão de consumo de álcool nesta população, com alta validade, confiabilidade (alpha de Cronbach > 0,80) e precisão (sensibilidade >0,80; especificidade > 0,70)^(10)^. É um instrumento composto por 10 questões, em que cada quesito varia numa escala entre 0 e 4 pontos, podendo resultar ao final do questionário completo em uma somatória entre 0 a 40 pontos^(11)^. De acordo com a pontuação obtida no instrumento, pode-se atribuir quatro classificações para o padrão de consumo de álcool, Zona I: consumo de baixo risco ou abstêmios (entre 0 e 7 pontos); Zona II: consumo de risco (entre 8 e 15 pontos); Zona III: consumo nocivo (entre 16 e 19 pontos) e Zona IV: provável dependência (entre 20 e 40 pontos).

Além do AUDIT, aplicou-se um questionário com perguntas relacionadas às características sociodemográficas: idade, raça/cor, sexo, estado civil, escolaridade, ocupação, renda familiar, estilo de vida, prática de atividade física, comorbidades, antecedentes clínicos e psiquiátricos. Também foram investigadas questões relacionadas à COVID-19, se o participante havia sido contaminado no período da pandemia, se houve necessidade de internação hospitalar e em Unidade de Terapia Intensiva (UTI), se perdeu familiares em decorrência da doença e se percebeu mudança no padrão de consumo de álcool em decorrência do período pandêmico.

Entende-se que as questões relacionadas à COVID-19 se justificam, uma vez que estudos prévios pontuam o aumento no consumo de álcool durante a pandemia como forma de enfrentamento, tanto às questões sociais, quanto emocionais, devido às internações, perda de familiares e dificuldades na elaboração do luto devido à ausência de rituais funerários adequados e ao confinamento prolongado^(12-13)^.

### Coleta de dados

A equipe de pesquisa teve acesso ao contato (número telefônico) dos potenciais participantes nas agendas dos serviços. Os gerentes das unidades de saúde enviavam as agendas mensalmente para o e-mail do grupo de pesquisa, a partir disso, os contatos telefônicos eram distribuídos entre os coletores da pesquisa, que realizavam cinco tentativas, em horários distintos, de contato aos usuários maiores de 18 anos, convidando-os a participar do estudo. Para aqueles que aceitaram participar, durante a chamada gravada explicava-se o motivo da pesquisa e seus objetivos, realizando a leitura do Termo de Consentimento Livre Esclarecido (TCLE), sendo disponibilizada uma cópia do termo via *WhatsApp* para o participante.

### Análise dos dados

As variáveis expositivas deste estudo foram organizadas de maneira hierarquizada das mais proximais (inatas do indivíduo) para aquelas mais distais (contextuais), em três níveis hierárquicos distintos. Nível 1: sexo (masculino e feminino), idade (anos contínuos), raça/cor (preta; parda, branca; oriental, indígena ou não declarada). Nível 2 (demográficas): status marital (solteiro; casado; amasiado/mora junto; divorciado/separado; viúvo), escolaridade (analfabeto; fundamental incompleto; fundamental completo; ensino médio incompleto; ensino médio completo; ensino superior incompleto, ensino superior completo; pós-graduação incompleta; pós-graduação completa; não sabe), renda familiar (< 1 salário-mínimo; de 1 a 5 salários-mínimos; de 6 a 10 salários-mínimos; > 10 salários-mínimos; não sabe). Nível 3 (saúde): realiza atividade física (sim; não), hipertensão arterial (sim; não); diabetes (sim; não); colesterol (sim; não); problemas gástricos (sim; não); depressão (sim; não).

As variáveis foram tratadas de maneira descritiva e inferencial por meio do *software IBM SPSS Statistics*. Variáveis categóricas foram descritas por meio de frequências absoluta (n) e relativa (%), enquanto a única variável contínua foi descrita por meio de média e desvio-padrão. A análise inferencial foi realizada por meio da Regressão de Poisson robusta para cálculo das Razões de Prevalência (RP), valor de p e intervalo de confiança de 95% (IC95%).

O modelo hierarquizado foi utilizado como base para as análises estatísticas multivariadas. No primeiro nível, todas as variáveis foram incluídas simultaneamente e ajustadas entre si. A partir do segundo nível, o modelo incluiu as variáveis do nível anterior que apresentaram valor de p < 0,20 na análise bivariada, além das novas variáveis do nível em questão, ajustadas entre si e em relação às variáveis mantidas dos níveis anteriores. Esse procedimento foi repetido para o terceiro nível. Optou-se por excluir as variáveis menos significativas de cada nível, visando a elaboração de modelos finais mais parcimoniosos, menos complexos e melhor ajustados. Essa abordagem oferece vantagens, como o controle de potenciais variáveis de confusão e a redução do risco de ajustes inadequados para variáveis que possam atuar como mediadoras na relação entre as variáveis expositivas e o desfecho^(14)^.

Para testagem da linearidade da variável idade, esta passou por transformação de potência 0,5. Os modelos com a variável original foram comparados com modelos nos quais a variável transformada foi incluída. Modelos sem a transformação demonstraram melhor ajuste, com menor Critério de Informação de Akaike corrigido e *deviance*. Portanto, a idade foi mantida como variável contínua sem transformação nos modelos adotados.

### Procedimentos éticos

O estudo foi aprovado pelo Comitê de Ética em Pesquisa, sob o parecer nº 4.342.492. Todos os indivíduos participaram mediante a concordância com o TCLE.

## Resultados

### Características da amostra

**Figura 1-:** Fluxograma de elegibilidade dos participantes. São Paulo, SP, Brasil, 2023

Da amostra, 69,8% eram do sexo feminino, apresentando idade média de 44,9 anos e 42,2% declararam raça/cor branca. Em relação ao status marital, 36,5% declararam-se como solteiros(as), enquanto, em relação à escolaridade, 36,3% declararam possuir ensino médio completo. A maioria da amostra declarou possuir renda familiar entre 1 e 5 salários-mínimos (64,8%). Em relação às características de saúde, 48,0% declararam realizar alguma atividade física, 31,0% reportaram hipertensão arterial, 15,1% diabetes, 19,2% colesterol, 27,0% problemas gástricos e 17,6% depressão. Sobre variáveis contextuais à pandemia de COVID-19, 33,6% afirmaram terem sido diagnosticados com a doença, 1,9% necessitaram de internação por COVID-19 e 0,7% internação em UTI por complicações da doença, enquanto 42,4% dos sujeitos declararam ter perdido alguém para a COVID-19.

Em relação aos padrões de consumo de álcool na amostra, 17,1% apresentaram consumo de risco e nocivo (AUDIT de 8 a 19 pontos), enquanto 1,8% da amostra se caracterizou como possível dependência para o consumo de álcool (AUDIT ≥ 20 pontos). Do total da amostra, 18,9% preencheram critérios para o consumo prejudicial de álcool, conforme a Tabela 1.

**Tabela 1-:** Prevalência de grupos de risco de uso de álcool em usuários da Atenção Primária à Saúde estratificada por sexo (n = 373). São Paulo, SP, Brasil, 2023

**Sexo**	**Consumo de risco/nocivo**			**Provável dependência**		
	n*	**Prevalência**	^†^ **IC 95%**	n*	**Prevalência**	^†^ **IC 95%**
Homens	156	7,2%	6,1; 8,2%	16	0,7%	0,4; 1,1%
Mulheres	217	9,9%	8,7; 11,2%	24	1,1%	0,7; 1,5%
Total	373	17,1%	15,7; 18,3%	40	1,8%	1,3; 2,4%

*n = Amostra; ^†^IC = Intervalo de confiança

Realizou-se uma análise multivariada hierarquizada para características associadas ao consumo de risco e nocivo do álcool. Nesta análise identificou-se que consumo de risco/nocivo de álcool foi mais prevalente entre o sexo masculino quando comparados com mulheres (RP = 1,89; IC95% 1,57 - 2,28; p < 0,001), entre pessoas que se declararam pretas quando comparadas com brancas (RP = 1,65; IC95% 1,30 - 2,10; p < 0,001), entre pessoas com renda ≥ 10 salários-mínimos quando comparadas com que recebe <1 salário-mínimo (RP = 1,78; IC95% 1,07 – 2,99; p = 0,028). Além disso, evidencia-se que o desfecho se torna menos prevalente com o avançar da idade (RP = 0,98; IC95% 0,97 – 0,99; p < 0,001), conforme Tabela 2.

**Tabela 2-:** Análise multivariada hierarquizada para características associadas ao consumo de risco e nocivo de álcool em usuários da Atenção Primária à Saúde (n = 373). São Paulo, SP, Brasil, 2023

	**Análise hierarquizada**									
	**Modelo 1**			**Modelo 2**					**Modelo 3**	
**Nível 1**	RP*	Valor de p ^†^	IC ^‡^ 95%	RP*	Valor de p ^†^	IC ^‡^ 95%		RP*	Valor de p ^†^	IC ^‡^ 95%
Sexo	**p <0,001** ^§^			**p <0,001** ^§^				**p <0,001** ^§^		
Masculino	1,84	<0,001	1,53; 2,21	1,82	<0,001	1,51; 2,19		1,89	<0,001	1,57; 2,28
Feminino	1			1				1		
Idade	**<0,001**			**<0,001**				**<0,001**		
Anos contínuos	0,98	<0,001	0,97; 0,99	0,97	<0,001	0,97; 0,98		0,98	<0,001	0,97; 0,99
Raça/cor	**p =0,002**			**p =0,004**				**p =0,002**		
Não declarou	1,47	0,239	0,77; 2,79	1,39	0,306	0,74; 2,59		1,42	0,292	0,74; 2,72
Preta	1,65	<0,001	1,30; 2,10	1,62	<0,001	1,26; 2,07		1,65	<0,001	1,30; 2,10
Parda	1,09	0,423	0,88; 1,36	1,09	0,444	0,87; 1,38		1,10	0,381	0,89; 1,37
Oriental	1,18	0,600	0,64; 2,16	1,18	0,595	0,64; 2,17		1,13	0,686	0,62; 2,08
Indígena	0,70	0,592	0,19; 2,56	0,71	0,597	0,20; 2,55		0,69	0,567	0,19; 2,47
Branca	1			1				1		
**Nível 2**										
Status marital				p =0,244			
Viúvo				1,19	0,604	0,62; 2,26				
Solteiro				1,26	0,075	0,98; 1,67				
Amasiado/Mora junto				1,23	0,174	0,91; 1,65				
Divorciado/Separado				1,46	0,034	1,03; 2,07				
Casado				1						
Escolaridade				p =0,763			
Fundamental completo				1,07	0,745		0,69; 1,67			
Fundamental incompleto				1,16	0,392		0,82; 1,65			
Ensino Médio completo				0,91	0,502		0,69; 1,20			
Ensino Médio incompleto				1,22	0,280		0,85; 1,77			
Ensino Superior incompleto				0,94	0,742		0,67; 1,33			
Pós-graduação completo				1,09	0,720		0,68; 1,76			
Pós-graduação incompleto				0,89	0,780		0,41; 1,94			
Não sabe				0,82	0,846		0,12; 5,86			
Ensino Superior completo				1						
Renda familiar				p =0,189					p =0,139	
De 1 a 5 SM ^||^				1,14	0,387		0,85; 1,54	1,13	0,416	0,84; 1,51
De 6 a 10 SM ^||^				1,28	0,280		0,81; 2,01	1,24	0,334	0,80; 1,90
≥ 10 SM ^||^				1,83	0,028		1,07; 3,16	1,78	0,028	1,07; 2,99
Não sabe informar				0,99	0,967		0,67; 1,47	0,53	0,751	0,64; 1,38
< 1 SM ^||^				1					1	
**Nível 3**									
Realiza atividade física								p =0,141		
Não								1,15	0,141	0,95; 1,38
Sim								1		
Hipertensão arterial								p =0,084		
Não								1,28	0,084	0,97; 1,63
Sim								1		
Diabetes								p =0,393		
Não								1,17	0,393	0,82; 1,67
Sim								1		
Colesterol								p =0,075		
Não								1,33	0,075	0,97; 1,82
Sim								1		
Problemas gástricos								p =0,378		
Não								0,91	0,378	0,74; 1,12
Sim								1		
Depressão								p =0,100		
Sim								1,20	0,100	0,96; 1,51
Não								1		
Critério de Informação de Akaike corrigido	1934,451			1951,693				1934,412		

Nota: Modelo 1 (ajustado para todas as variáveis do Nível 1); Modelo 2 (ajustado para as variáveis com p<0,200 no teste de efeito global do nível acima e para todas as variáveis do Nível 2); Modelo 3 (ajustado para as variáveis com p<0,200 no teste de efeito global dos níveis acima e para todas as variáveis do Nível 3; A categoria “analfabeto” foi retirada da análise pela baixa frequência de indivíduos com o desfecho e gerar problemas de singularidade de matriz hessiana no modelo. O Critério de Informação de Akaike (AIC) avalia modelos estatísticos equilibrando o ajuste aos dados com a complexidade dos parâmetros. Modelos com menor AIC são preferíveis, pois evitam sobre ajuste sem perder desempenho. *RP = Razão de prevalência; ^†^Valor de p = Nível de significância; ^‡^IC = Intervalo de confiança; ^§^p <0,001 = Significância estatística; ^||^SM = Salário-mínimo, vigente no Brasil em 2023, R$ 1.320,00

Na Tabela 2, o modelo 2 apresentou o pior desempenho (AICc = 1951,693), sugerindo que a adição das variáveis demográficas piorou o ajuste, provavelmente por não contribuírem significativamente para explicar o desfecho. Por sua vez, o modelo 3 apresentou o menor AICc (1934,412), indicando o melhor equilíbrio entre ajuste e complexidade representada pelo número de variáveis incluídas.

A análise multivariada hierarquizada para características associadas à provável dependência de álcool (AUDIT ≥ 20 pontos) evidenciou que este desfecho foi mais prevalente entre usuários com menor nível de escolaridade (RP = 13,70; IC95% 1,56 – 117,63; p = 0,017) e que declararam ensino superior incompleto (RP = 4,10; IC95% 1,09 – 15,42; p = 0,037) do que aqueles com ensino superior completo. Também foi mais prevalente entre usuários com depressão do que aqueles que negaram a patologia (RP = 2,72; IC95% 1,37 – 5,41; p = 0,005), conforme a Tabela 3.

**Tabela 3-:** Análise multivariada hierarquizada para características associadas à provável dependência de álcool em usuários da atenção primária à saúde (n = 40). São Paulo, SP, Brasil, 2023

	**Análise hierarquizada**									
	**Modelo 1**			**Modelo 2**			**Modelo 3**			
**Nível 1**	RP*	Valor de p ^†^	IC ^‡^ 95%	RP*	Valor de p ^†^	IC ^‡^ 95%	RP*	Valor de p ^†^	IC ^‡^ 95%
Sexo	p =0,174 ^§^			p =0,153 ^§^			p =0,106 ^§^			
Masculino	1,58	0,174	0,82; 3,05	1,59	0,153	0,84; 3,01	1,75	0,106	0,89; 3,45
Feminino	1			1			1		
Idade	**p =0,030**			**p =0,006**			p =0,269			
Anos contínuos	0,98	0,030	0,96; 1,00	0,97	0,006	0,96; 0,99	0,99	0,269	0,97; 1,01
Raça/cor	p =0,774								
Não declarou	1,89	0,534	0,25; 13,94						
Preta	0,94	0,904	0,33; 2,63						
Parda	1,34	0,416	0,66; 2,73						
Oriental	1,44	0,720	0,19; 10,63						
Indígena	3,51	0,203	0,51; 24,34						
Branca	1								
**Nível 2**										
Status marital				p =0,208					
Viúvo				2,58	0,224	0,56; 11,89			
Solteiro				1,43	0,434	0,58; 3,52			
Amasiado/Mora junto				1,33	0,587	0,48; 3,68			
Divorciado/Separado				3,31	**0,021**	1,20; 9,13			
Casado				1					
Escolaridade				p =0,121			p =0,191			
Analfabeto				15,19	**0,015**	1,70; 135,98	13,70	**0,017**	1,60; 117,63
Fundamental completo				3,25	0,142	0,67; 15,66	3,07	0,171	0,62; 15,29
Fundamental incompleto				4,33	**0,025**	1,21; 15,54	3,88	0,051	0,99; 15,14
Ensino Médio completo				2,33	0,158	0,72; 7,55	2,21	0,214	0,63; 7,72
Ensino Médio incompleto				1,64	0,574	0,29; 9,24	1,42	0,694	0,24; 8,29
Ensino Superior incompleto				4,67	**0,020**	1,28; 17,11	4,10	**0,037**	1,09; 15,42
Pós-graduação completo				1,25	0,854	0,12; 13,48	1,26	0,842	0,13; 12,21
Ensino Superior completo				1			1		
Renda familiar				p =0,390					
De 1 a 5 SM ^||^				0,91	0,846	0,37; 2,24			
De 5 a 10 SM ^||^				2,09	0,234	0,62; 7,06			
≥ 10 SM ^||^				1,10	0,933	0,12; 9,70			
Não sabe informar				0,49	0,317	0,12; 1,99			
< 1 SM ^||^				1					
**Nível 3**										
Realiza atividade física							p =0,501			
Não							1,26	0,501	0,64; 2,48
Sim							1		
Hipertensão arterial							p =0,400			
Não							1,50	0,400	0,58; 3,82
Sim							1		
Diabetes							p =0,119			
Não							3,11	0,119	0,75; 12,97
Sim							1		
Colesterol							p =0,961			
Não							0,98	0,961	0,43; 2,23
Sim							1		
Problemas gástricos							p =0,851			
Não							0,94	0,851	0,47; 1,86
Sim							1		
Depressão							**p =0,005**			
Sim							2,72	0,005	1,37; 5,41
Não							1		
Critério de Informação de Akaike corrigido	399,086			406,284			397,777			

Nota: Modelo 1 (ajustado para todas as variáveis do Nível 1); Modelo 2 (ajustado para as variáveis com p<0,200 no teste de efeito global do nível acima e para todas as variáveis do Nível 2); Modelo 3 (ajustado para as variáveis com p<0,200 no teste de efeito global dos níveis acima e para todas as variáveis do Nível 3); As categorias “pós-graduação incompleta” e “não sabe” da variável escolaridade foram retiradas da análise pela baixa frequência de indivíduos com o desfecho e geraram problemas de singularidade de matriz hesiana no modelo. O Critério de Informação de Akaike (AIC) avalia modelos estatísticos equilibrando o ajuste aos dados com a complexidade dos parâmetros. Modelos com menor AIC são preferíveis, pois evitam sobreajuste sem perder desempenho. *RP = Razão de prevalência; ^†^Valor de p = Nível de significância; ^‡^IC= Intervalo de confiança; ^§^p <0,001 = Significância estatística; ^||^SM = Salário-mínimo, vigente no Brasil em 2023, R$ 1.320,00

Na Tabela 3, o modelo 2 apresentou o pior desempenho (AICc = 406,284), sugerindo que a adição das variáveis demográficas piorou o ajuste, provavelmente por não contribuírem significativamente para explicar o desfecho. Por sua vez, o modelo 3 apresentou o menor AICc (397,77), indicando o melhor equilíbrio entre ajuste e complexidade representada pelo número de variáveis incluídas.

## Discussão

O presente estudo objetivou identificar a prevalência dos padrões de consumo de álcool entre usuários da APS e a associação entre os padrões de consumo de risco, nocivo e provável dependência, com características sociodemográficas, clínicas e comportamentais. Os resultados sobre a prevalência de consumo de risco/nocivo no presente estudo foram de 17,1%, destoando de um levantamento no município de São Paulo que identificou a prevalência de 7,7% na população em geral^(15)^.

No recorte por sexo, as mulheres no presente estudo apresentaram prevalência de consumo de risco/nocivo de 9,9%, frente a 3,7% no levantamento municipal; já na população masculina foi de 7,2% e 12,4% respectivamente. Ressalta-se que 70% da amostra era composta por mulheres, corroborando estudo prévio^(16)^. O que, nesse contexto, pode refletir maior percepção sobre o próprio consumo, decorrente do acompanhamento contínuo na UBS e maior consciência sobre seu processo de saúde-doença.

Apesar disso, na análise multivariada o sexo masculino foi associado ao padrão de consumo de risco/nocivo. Entende-se que esse padrão está intimamente ligado a fatores culturais e à atribuição de papéis sociais distintos. Enquanto o consumo de bebidas alcoólicas por homens pode ser associado à expressão de masculinidade e sociabilidade, as mulheres frequentemente enfrentam normas sociais mais restritivas, que desencorajam o mesmo comportamento^(17)^.

Os achados apresentam evidências claras de consumo de risco e nocivo entre jovens, compatível com pesquisa anterior que destaca uma preocupante associação entre o consumo precoce de bebidas alcoólicas e a adoção de outros comportamentos de risco^(18)^. Entende-se que essa alta prevalência de uso de álcool entre jovens associa-se à fácil acessibilidade à substância, somada às influências sociais e culturais, contribuindo para a banalização do seu consumo^(19-20)^.

O consumo de risco/nocivo também foi associado às pessoas que se autodeclararam como pretas, revelando a intersecção entre racismo estrutural e saúde mental, na qual indivíduos são diagnosticados a partir da cor de sua pele, além de terem maiores dificuldades de acesso aos serviços de saúde^(21-23)^. O estudo realizado com veteranos identificou que homens negros tinham de 23% a 109% mais chances de receber um diagnóstico de transtorno por uso de álcool em relação a homens brancos, evidenciado o viés de diagnóstico a qual as pessoas negras estão submetidas^(21)^. Além disso, estudos destacam o menor acesso aos cuidados em saúde da população negra em relação à população branca, enfatizando as consequências negativas nos resultados de saúde dessa população^(22-23)^.

Entende-se que desigualdades são perpetuadas por barreiras institucionais e experiências de discriminação nos serviços de saúde, onde determinados grupos sociais são sistematicamente excluídos e marginalizados nas políticas de cuidado em álcool e outras drogas, reproduzindo violências históricas e institucionais^(24-26)^. O cenário demanda uma reformulação urgente das políticas públicas de saúde mental e da formação profissional, incorporando uma perspectiva antirracista e culturalmente sensível que reconheça e enfrente ativamente os marcadores sociais das desigualdades. Tal abordagem é fundamental para desenvolver estratégias de cuidado que promovam equidade e rompam com os ciclos de exclusão e estigmatização.

Apesar de ter sido encontrado no estudo uma associação do consumo de risco/nocivo de álcool por pessoas com maior renda, a mesma revela-se complexa. Estudos apontam que indivíduos de baixa renda tendem a consumir álcool de forma mais frequente e intensa, em resposta às adversidades de fatores socioeconômicos e psicológicos^(24,27)^, em contrapartida, pessoas de alta renda apresentam consumo elevado, impulsionado por fatores culturais e sociais, como eventos e demonstração de status^(28-29)^. Além disso, o acesso a cuidados e suporte em saúde é maior entre os indivíduos de alta renda, o que pode agravar os desfechos para os economicamente vulneráveis, que enfrentam barreiras significativas para buscar tratamento^(30)^.

Ressalta-se ainda que a pandemia intensificou esses desafios. Estudos apontam implicações importantes para a acessibilidade ao consumo devido a impactos financeiros, além de políticas públicas de controle do consumo^(31)^, e embora não tenha sido observada uma associação significativa entre o consumo prejudicial de álcool e os impactos diretos da pandemia na amostra analisada, é importante destacar que a literatura aponta um aumento generalizado nos comportamentos de risco à saúde durante o período de isolamento social^(32)^.

Esse cenário sugere que o contexto de crise sanitária pode ter exacerbado fatores de risco para o uso problemático de álcool em outras populações, tornando-se imprescindível implementar medidas preventivas na APS, considerando as múltiplas dimensões que influenciam os padrões de consumo e sua relação com os fatores socioeconômicos da população.

As comorbidades mais autorreferidas foram: hipertensão, diabetes mellitus, gastrite e colesterol. Apesar da ausência de significância estatística, os dados corroboram com a literatura, que aponta uma alta prevalência de doenças crônicas preexistentes entre indivíduos que fazem uso prejudicial de álcool na APS, resultando no agravamento de tais condições^(33-34)^. A falta de abordagem sistemática do consumo de álcool contribui para subdiagnóstico e na ausência de prevenção e tratamento precoce dos transtornos relacionados ao álcool^(35)^, ressaltando a necessidade de maior atenção ao rastreamento e às intervenções na APS.

É importante destacar que, por se tratar de um estudo transversal, não é possível estabelecer relações causais ou temporais entre o consumo de álcool e as comorbidades identificadas. A complexidade dessas associações pode extrapolar os limites de um estudo transversal, uma vez que fatores socioculturais, econômicos e comportamentais, que não foram incluídos no modelo estatístico, podem desempenhar papéis importantes nessas associações, sendo recomendáveis estudos longitudinais que incorporem uma gama mais ampla de variáveis para elucidar tais questões, sugestões para estudos futuros.

Os resultados deste estudo apontaram associação entre nível de escolaridade, depressão autodeclarada e provável dependência de álcool, alinhando-se a estudos prévios^(20,36)^. Entende-se que a educação desempenha um papel crucial no desenvolvimento de habilidades de enfrentamento e na formação de redes de suporte social, fatores que contribuem para a prevenção da dependência, por outro lado, sua ausência pode limitar o acesso a essas ferramentas protetoras, favorecendo comportamentos de risco^(37-38)^. Considerando que o álcool é frequentemente utilizado como uma estratégia para aliviar os sintomas de sofrimento mental, como ansiedade e tristeza, destaca-se a necessidade de abordagens integradas que considerem tanto os aspectos psicológicos quanto os sociais para o melhor encaminhamento dos casos^(22-34)^.

Conforme evidenciado pelos resultados deste estudo, os problemas relacionados ao consumo de álcool são influenciados tanto por fatores individuais quanto por determinantes sociais. Portanto, com seu caráter integral e territorializado, a APS apresenta-se como um espaço estratégico para ações de promoção da saúde, prevenção de agravos e redução de danos. No entanto, para que as estratégias sejam efetivas, é imprescindível superar as barreiras organizacionais existentes, como a alta demanda de cuidados e a sobrecarga dos profissionais, além de investir na capacitação das equipes para uma abordagem livre de estigmas e centrada no acolhimento.

Este estudo deve ser interpretado no contexto de limitações, dentre elas que todos os resultados são baseados em autorrelato, o que pode levar à omissão de informações ou à subestimação da frequência e quantidade de álcool consumido, limitações de acesso a serviços especializados, devido a possíveis vieses de desejabilidade social. Foram realizadas estratégias na padronização da coleta de dados e alinhamento dos pesquisadores para minimizar tal viés.

Ademais, o delineamento transversal também limita a inferência causal, uma vez que não é possível determinar a temporalidade entre a exposição (consumo de álcool) e os desfechos (como comorbidades e transtornos mentais). A coleta durante a pandemia pode ter introduzido viés de memória, além da falta de dados comparativos pré-pandemia, limitando a capacidade de avaliar mudanças específicas nesse período. Ainda, a amostra, por não ser representativa do município ou de outras regiões, impossibilita a generalização dos dados.

Outrossim, variáveis como religião/religiosidade, suporte social, outros fatores protetores conhecidos e a exclusão de pessoas surdas não foram consideradas podem representar uma limitação adicional. Contudo, os achados deste estudo podem subsidiar futuras pesquisas sobre prevalência dos padrões de consumo de álcool entre os usuários dos serviços de APS, que considerem os aspectos elencados.

## Conclusão

Conclui-se que o estudo, ao buscar identificar a prevalência dos padrões de consumo de álcool entre usuários da APS na cidade de São Paulo, apontou que o seu consumo prejudicial está associado principalmente a usuários do sexo masculino, mais jovens, de raça/cor preta e com renda igual ou superior a 10 salários mínimos. A busca por atendimento na APS ocorre em sua maioria, por outros problemas de saúde, o que reforça a importância desse nível de atenção como um cenário estratégico para a identificação precoce do consumo prejudicial de álcool e a implementação de medidas preventivas.

## Data Availability

Todos os dados gerados ou analisados durante este estudo estão incluídos neste artigo publicado.

# Translation: Factores de riesgo para el consumo perjudicial de alcohol: análisis multinivel*

**DOI:** 10.1590/1518-8345.7763.4685

**Authors:** Jéssica Lima de Oliveira Pinheiro Gaia, Ana Vitória Corrêa Lima, Sheila Ramos de Oliveira, Jaqueline Lemos de Oliveira, Caroline Figueira Pereira, Divane de Vargas

**Affiliations:** 1 Escola de Enfermagem da Universidade de São Paulo, Departamento Materno-Infantil e Psiquiátrica, São Paulo, SP, Brasil; 2 Becaria de la Coordenação de Aperfeiçoamento de Pessoal de Nível Superior (CAPES), Brasil; 3 Becaria del Ministério da Saúde, Instituto de Psiquiatria da USP, Brasil

**Keywords:** Salud, Prevalencia, Salud Mental, Atención Primaria de Salud, Trastornos Inducidos por Alcohol, Consumidores de Drogas

## Abstract

identificar la prevalencia de los patrones de consumo de alcohol entre usuarios de la Atención Primaria de Salud y la asociación entre los patrones de consumo de riesgo, nocivo y probable dependencia con características sociodemográficas, clínicas y conductuales.

estudio transversal, realizado con una muestra de 2178 participantes que respondieron al *Alcohol Use Disorders Identification Test*, en servicios de Atención Primaria de Salud del municipio de São Paulo. Se realizó un análisis estadístico descriptivo e inferencial múltiple con modelo jerarquizado de regresión de Poisson con un nivel de significancia del 5%.

del total de participantes, el 18,9% cumplió criterios para consumo perjudicial de alcohol. Fueron determinantes para el consumo de riesgo y nocivo de alcohol el sexo masculino (RP = 1,89; IC95% 1,57 - 2,28; p < 0,001), raza/color negra (RP = 1,65; IC95% 1,30 - 2,10; p < 0,001) e ingresos ≥ 10 salarios mínimos (RP = 1,78; IC95% 1,07 – 2,99; p = 0,028). Para la probable dependencia fueron determinantes un menor nivel educativo (RP = 13,70; IC95% 1,56 – 117,63; p = 0,017) y referir diagnóstico de depresión (RP = 2,72; IC95% 1,37 – 5,41; p = 0,005).

la prevalencia de trastornos por consumo de alcohol entre usuarios de los servicios de atención primaria de salud de São Paulo fue superior a la observada en estudios previos, lo que sugiere que esta parte de la población busca atención en estos servicios, destacando el potencial para el cribado y diagnóstico precoz de la población.

## Destaques

**(1)** Alta prevalencia de trastornos por uso de alcohol en la Atención Primaria de Salud. **(2)** Factores de riesgo: sexo, raza/color, ingresos, bajo nivel educativo y depresión. **(3)** Necesidad de estrategias de cribado e intervención.

## Introducción

El consumo perjudicial de alcohol está asociado a daños tanto para la propia persona como para terceros, tales como familiares, amigos, compañeros de trabajo o desconocidos. Esto ocurre porque incrementa directamente el riesgo de desarrollar problemas de salud como enfermedades cardiovasculares y hepáticas, así como de presentar conductas de riesgo que pueden derivar en violencia y accidentes de tráfico ^(1 )^ . Además, el alcohol impacta negativamente la salud mental y la calidad de vida de los individuos, hecho que se observó de forma aún más intensa durante la pandemia de COVID-19 ^(2 )^ .

Brasil se encuentra entre los países de las Américas con los índices más altos de consumo de alcohol per cápita. Según información del Observatorio de Salud Pública, el 35,4% de la población brasileña consume bebidas alcohólicas, con una mayor prevalencia entre adultos residentes en las capitales brasileñas, que consumen la sustancia al menos una vez por semana, conforme lo revelan los datos de la Vigilancia de Factores de Riesgo y Protección para Enfermedades Crónicas mediante Encuesta Telefónica (Vigitel) ^(3 )^ .

La Atención Primaria de Salud (APS) se configura como un espacio importante para el cribado e identificación del consumo perjudicial de alcohol, ya que constituye la puerta de entrada de la población a los servicios de salud, alcanzando una tasa de cobertura del 83% de la población, considerando el promedio nacional ^(4 )^ . Además, existen evidencias de que cerca del 20% de las personas que reciben tratamiento por morbilidades en la APS refieren un consumo perjudicial de alcohol ^(5-6 )^ . Entre los cuadros clínicos más comunes en la APS, frecuentemente asociados al consumo perjudicial de alcohol, se encuentran hipertensión, insomnio, problemas hepáticos, depresión y ansiedad ^(7 )^ .

En este contexto, la APS demuestra un gran potencial para el cribado y diagnóstico precoz de los usuarios que presentan un consumo perjudicial de alcohol, debido a su facilidad de acceso, la capacidad de establecer vínculos y su enfoque integral, que incluye acciones de promoción de la salud y prevención de agravios, consolidándose como un dispositivo esencial de cuidado ^(6 )^ .

A pesar de estas potencialidades, existen barreras que dificultan e incluso impiden que ocurra una búsqueda efectiva de estos pacientes en dichos escenarios. En general, los desafíos están marcados principalmente por dificultades organizacionales en las instituciones, relacionadas con altas demandas de atención, la carga del trastorno asociada a la escasez de intervenciones debidamente implementadas en la APS, el estigma y los prejuicios enfrentados, además de la baja prevalencia de usuarios que tienen su consumo de alcohol evaluado por los profesionales de salud ^(8-9 )^ . Tales factores justifican la implementación de estrategias de cribado en la APS, para que se realice la identificación precoz del riesgo de consumo perjudicial de alcohol y se establezcan intervenciones eficaces para quienes hacen un uso abusivo de la sustancia. Se entiende que estos patrones se presentan como un factor de riesgo para la salud de la población, especialmente para quienes presentan otras enfermedades crónicas asociadas.

En este sentido, considerando que son escasas las investigaciones brasileñas que se han ocupado de identificar el patrón de consumo de alcohol de individuos que buscan atención en servicios de APS, este estudio tiene como objetivo identificar la prevalencia de los patrones de consumo de alcohol entre usuarios de la APS y la asociación entre los patrones de consumo de riesgo, nocivo y probable dependencia con características sociodemográficas, clínicas y conductuales.

## Método

### Diseño del estudio y participantes

Estudio transversal, realizado entre noviembre de 2021 y mayo de 2023, con usuarios mayores de 18 años atendidos en Unidades Básicas de Salud (UBS) del municipio de São Paulo. La recolección de datos se llevó a cabo en 13 de las 479 UBS del municipio de São Paulo. Las unidades seleccionadas están distribuidas en diferentes regiones de la ciudad, incluyendo: una del Norte, dos del Sur, dos del Sudeste, dos del Este, dos del Oeste y cuatro de la región Central. A pesar de tratarse de una muestra por conveniencia, la selección procuró considerar la diversidad geográfica y sociodemográfica, con representantes de cada región del municipio. Los posibles participantes fueron reclutados utilizando la agenda diaria de atenciones de cada unidad. Los criterios de exclusión fueron: presentar un patrón de bajo riesgo/abstinencia, estar en tratamiento por problemas relacionados al consumo de alcohol, ser persona con discapacidad auditiva y presentar señales perceptibles de confusión mental o intoxicación por sustancias psicoactivas durante la recolección de datos. Para evaluar los signos de confusión mental se realizaron preguntas sobre el día de la semana, el mes, el año, así como la identificación del momento del día en que se realizaba la entrevista –mañana, tarde o noche– según el horario correspondiente. Para evaluar signos de intoxicación por sustancias psicoactivas se incluyó la evaluación del habla del usuario, observando si era pastosa, y la coherencia de las respuestas.

### Instrumentos para la recolección de datos

El instrumento *Alcohol Use Disorders Identification Test* (AUDIT), utilizado exclusivamente para la identificación de problemas asociados al consumo de alcohol, fue validado para la población brasileña en un estudio con pacientes atendidos en el contexto de la APS, identificándose como un instrumento útil para el cribado del patrón de consumo de alcohol en esta población, con alta validez, confiabilidad (alfa de Cronbach > 0,80) y precisión (sensibilidad > 0,80; especificidad > 0,70) ^(10 )^ . Se trata de un instrumento compuesto por 10 preguntas, en las que cada ítem varía en una escala de 0 a 4 puntos, pudiendo resultar al final del cuestionario completo en una sumatoria entre 0 y 40 puntos ^(11 )^ . De acuerdo con la puntuación obtenida en el instrumento, se pueden asignar cuatro clasificaciones para el patrón de consumo de alcohol: Zona I: consumo de bajo riesgo o abstemios (entre 0 y 7 puntos); Zona II: consumo de riesgo (entre 8 y 15 puntos); Zona III: consumo nocivo (entre 16 y 19 puntos) y Zona IV: probable dependencia (entre 20 y 40 puntos).

Además del AUDIT se aplicó un cuestionario con preguntas relacionadas a las características sociodemográficas: edad, raza/color, sexo, estado civil, nivel educativo, ocupación, ingreso familiar, estilo de vida, práctica de actividad física, comorbilidades, antecedentes clínicos y psiquiátricos. También se investigaron cuestiones relacionadas con la COVID-19, como si el participante había sido contagiado durante el período de la pandemia, si fue necesaria la hospitalización o ingreso en Unidad de Cuidados Intensivos (UCI), si perdió familiares a causa de la enfermedad y si percibió un cambio en el patrón de consumo de alcohol como consecuencia del período pandémico.

Se entiende que las cuestiones relacionadas con la COVID-19 se justifican, ya que estudios previos indican que hubo un aumento en el consumo de alcohol durante la pandemia como forma de afrontamiento tanto de cuestiones sociales como emocionales, debido a hospitalizaciones, pérdida de familiares y dificultades para elaborar el duelo ante la ausencia de rituales funerarios adecuados y al confinamiento prolongado ^(12-13 )^ .

### Recolección de datos

El equipo de investigación tuvo acceso al contacto (número telefónico) de los potenciales participantes por medio las agendas de los servicios. Los gerentes de las unidades de salud enviaban las agendas mensualmente al correo electrónico del grupo de investigación, y a partir de ello, los contactos telefónicos eran distribuidos entre los encuestadores, quienes realizaban cinco intentos, en horarios distintos, para contactar a los usuarios mayores de 18 años, invitándolos a participar del estudio. Para aquellos que aceptaban participar, durante la llamada grabada se explicaba el motivo de la investigación y sus objetivos, realizando la lectura del Consentimiento Informado (CI), y se enviaba una copia del término vía *WhatsApp* al participante.

### Análisis de datos

Las variables explicativas de este estudio fueron organizadas de manera jerarquizada, desde las más proximales (innatas del individuo) hasta las más distales (contextuales), en tres niveles jerárquicos distintos. Nivel 1: sexo (masculino y femenino), edad (años continuos), raza/color (negra; parda, blanca; oriental, indígena o no declarada). Nivel 2 (demográficas): estado civil (soltero; casado; en unión libre; divorciado/separado; viudo), nivel educativo (analfabeto; educación primaria incompleta; primaria completa; secundaria incompleta; secundaria completa; educación superior incompleta; superior completa; posgrado incompleto; posgrado completo; no sabe), ingreso familiar (< 1 salario mínimo; de 1 a 5 salarios mínimos; de 6 a 10 salarios mínimos; > 10 salarios mínimos; no sabe). Nivel 3 (salud): realiza actividad física (sí; no), hipertensión arterial (sí; no); diabetes (sí; no); colesterol (sí; no); problemas gástricos (sí; no); depresión (sí; no).

Las variables fueron tratadas de manera descriptiva e inferencial mediante el *software IBM SPSS Statistics* . Las variables categóricas se describieron mediante frecuencias absolutas (n) y relativas (%), mientras que la única variable continua se describió mediante media y desviación estándar. El análisis inferencial se realizó mediante Regresión de Poisson robusta para el cálculo de las Razones de Prevalencia (RP), valor de *p* e intervalo de confianza del 95% (IC95%).

El modelo jerarquizado se utilizó como base para los análisis estadísticos multivariados. En el primer nivel, todas las variables fueron incluidas simultáneamente y ajustadas entre sí. A partir del segundo nivel, el modelo incluyó las variables del nivel anterior que presentaron un valor de p < 0,20 en el análisis bivariado, además de las nuevas variables del nivel en cuestión, ajustadas entre sí y en relación con las variables mantenidas de los niveles anteriores. Este procedimiento se repitió para el tercer nivel. Se optó por excluir las variables menos significativas de cada nivel, con el objetivo de elaborar modelos finales más parsimoniosos, menos complejos y mejor ajustados. Este enfoque ofrece ventajas, como el control de posibles variables de confusión y la reducción del riesgo de ajustes inadecuados para variables que puedan actuar como mediadoras en la relación entre las variables explicativas y el desenlace ^(14 )^ .

Para la prueba de linealidad de la variable edad, esta fue sometida a una transformación de potencia 0,5. Los modelos con la variable original se compararon con modelos en los que se incluyó la variable transformada. Los modelos sin la transformación demostraron un mejor ajuste, con un menor Criterio de Información de Akaike corregido y *deviance* . Por tanto, la edad se mantuvo como variable continua sin transformación en los modelos adoptados.

### Procedimientos éticos

El estudio fue aprobado por el Comité de Ética en Investigación, bajo el dictamen nº 4.342.492. Todos los individuos participaron mediante la aceptación del CI.

## Resultados

### Características de la muestra

**Figura 1-:** Diagrama de flujo de elegibilidad de los participantes. São Paulo, SP, Brasil, 2023

De la muestra, el 69,8% eran del sexo femenino, presentaron una edad media de 44,9 años y el 42,2% se autodeclararon de raza/color blanca. Con respecto al estado civil, el 36,5% se declararon solteros(as), mientras que, en cuanto al nivel educativo, el 36,3% reportaron haber completado la enseñanza secundaria. La mayoría de la muestra declaró tener un ingreso familiar entre 1 y 5 salarios mínimos (64,8%). En relación con las características de salud, el 48,0% afirmaron realizar alguna actividad física, el 31,0% reportaron hipertensión arterial, el 15,1% diabetes, el 19,2% colesterol, el 27,0% problemas gástricos y el 17,6% depresión. Sobre las variables contextuales a la pandemia de COVID-19, el 33,6% afirmaron haber sido diagnosticados con la enfermedad, el 1,9% requirieron hospitalización por COVID-19 y el 0,7% ingreso en UCI por complicaciones de la enfermedad, mientras que el 42,4% de los sujetos declararon haber perdido a alguien a causa de la COVID-19.

En cuanto a los patrones de consumo de alcohol en la muestra, el 17,1% presentaron consumo de riesgo y nocivo (AUDIT de 8 a 19 puntos), mientras que el 1,8% de la muestra se caracterizó como posible dependencia al consumo de alcohol (AUDIT ≥ 20 puntos). Del total de la muestra, el 18,9% cumplió criterios para el consumo perjudicial de alcohol, conforme a la Tabla 1 .

**Tabla 1-:** Prevalencia de grupos de riesgo por uso de alcohol en usuarios de la Atención Primaria de Salud estratificada por sexo (n = 373). São Paulo, SP, Brasil, 2023

**Sexo**	**Consumo de riesgo/nocivo**			**Probable dependencia**		
	n*	**Prevalencia**	**IC** ^†^ **95%**	n*	**Prevalencia**	**IC** ^†^ **95%**
Hombres	156	7,2%	6,1; 8,2%	16	0,7%	0,4; 1,1%
Mujeres	217	9,9%	8,7; 11,2%	24	1,1%	0,7; 1,5%
Total	373	17,1%	15,7; 18,3%	40	1,8%	1,3; 2,4%

*n = Muestra; †IC = Intervalo de confianza

Se realizó un análisis multivariado jerarquizado para identificar características asociadas al consumo de riesgo y nocivo de alcohol. En este análisis se identificó que el consumo de riesgo/nocivo de alcohol fue más prevalente entre hombres en comparación con mujeres (RP = 1,89; IC95% 1,57 - 2,28; p < 0,001), entre personas que se autodeclararon negras en comparación con blancas (RP = 1,65; IC95% 1,30 - 2,10; p < 0,001), y entre personas con ingresos ≥ 10 salarios mínimos en comparación con aquellas que reciben < 1 salario mínimo (RP = 1,78; IC95% 1,07 – 2,99; p = 0,028). Además, se evidenció que el desenlace se vuelve menos prevalente con el avance de la edad (RP = 0,98; IC95% 0,97 – 0,99; p < 0,001), conforme a la Tabla 2 .

**Tabla 2-:** Análisis multivariado jerarquizado de características asociadas al consumo de riesgo y nocivo de alcohol en usuarios de la Atención Primaria de Salud (n = 373). São Paulo, SP, Brasil, 2023

	**Análisis jerarquizado**								
	**Modelo 1**			**Modelo 2**				**Modelo 3**	
**Nivel 1**	RP*	Valor de p ^†^	IC ^‡^ 95%	RP*	Valor de p ^†^	IC ^‡^ 95%	RP*	Valor de p ^†^	IC ^‡^ 95%
Sexo	**p <0,001** ^§^			**p <0,001** ^§^			**p <0,001** ^§		
Masculino	1,84	<0,001	1,53; 2,21	1,82	<0,001	1,51; 2,19	1,89	<0,001	1,57; 2,28
Femenino	1			1			1		
Edad	**<0,001**			**<0,001**			**<0,001**		
Años continuos	0,98	<0,001	0,97; 0,99	0,97	<0,001	0,97; 0,98	0,98	<0,001	0,97; 0,99
Raza/color	**p =0,002**			**p =0,004**			**p =0,002**		
No declaró	1,47	0,239	0,77; 2,79	1,39	0,306	0,74; 2,59	1,42	0,292	0,74; 2,72
Negra	1,65	<0,001	1,30; 2,10	1,62	<0,001	1,26; 2,07	1,65	<0,001	1,30; 2,10
Parda	1,09	0,423	0,88; 1,36	1,09	0,444	0,87; 1,38	1,10	0,381	0,89; 1,37
Oriental	1,18	0,600	0,64; 2,16	1,18	0,595	0,64; 2,17	1,13	0,686	0,62; 2,08
Indígena	0,70	0,592	0,19; 2,56	0,71	0,597	0,20; 2,55	0,69	0,567	0,19; 2,47
Blanca	1			1			1		
**Nivel 2**									
Estado civil				p =0,244					
Viudo				1,19	0,604	0,62; 2,26			
Soltero				1,26	0,075	0,98; 1,67			
En unión libre/Convive				1,23	0,174	0,91; 1,65			
Divorciado/Separado				1,46	0,034	1,03; 2,07			
Casado				1					
Escolaridad				p =0,763					
Educación primaria completa				1,07	0,745	0,69; 1,67			
Educación primaria incompleta				1,16	0,392	0,82; 1,65			
Educación secundaria completa				0,91	0,502	0,69; 1,20			
Educación secundaria incompleta				1,22	0,280	0,85; 1,77			
Educación superior incompleta				0,94	0,742	0,67; 1,33			
Posgrado completo				1,09	0,720	0,68; 1,76			
Posgrado incompleto				0,89	0,780	0,41; 1,94			
No sabe				0,82	0,846	0,12; 5,86			
Educación superior completa				1					
Ingresos familiares				p =0,189			p =0,139		
De 1 a 5 SM ^||^				1,14	0,387	0,85; 1,54	1,13	0,416	0,84; 1,51
De 6 a 10 SM ^||^				1,28	0,280	0,81; 2,01	1,24	0,334	0,80; 1,90
≥ 10 SM ^||^				1,83	0,028	1,07; 3,16	1,78	0,028	1,07; 2,99
No sabe informar				0,99	0,967	0,67; 1,47	0,53	0,751	0,64; 1,38
< 1 SM ^||^				1			1		
**Nivel 3**									
Realiza actividad física							p =0,141		
No							1,15	0,141	0,95; 1,38
Sí							1		
Hipertensión arterial							p =0,084		
No							1,28	0,084	0,97; 1,63
Sí							1		
Diabetes							p =0,393		
No							1,17	0,393	0,82; 1,67
Sí							1		
Colesterol							p =0,075		
No							1,33	0,075	0,97; 1,82
Sí							1		
Problemas gástricos							p =0,378		
No							0,91	0,378	0,74; 1,12
Sí							1		
Depresión							p =0,100		
Sí							1,20	0,100	0,96; 1,51
No							1		
Criterio de Información de Akaike corregido	1934,451			1951,693			1934,412		

Nota: Modelo 1 (ajustado para todas las variables del Nivel 1); Modelo 2 (ajustado para las variables con p < 0,200 en la prueba de efecto global del nivel anterior y para todas las variables del Nivel 2); Modelo 3 (ajustado para las variables con p < 0,200 en la prueba de efecto global de los niveles anteriores y para todas las variables del Nivel 3). La categoría “analfabeto” fue excluida del análisis debido a la baja frecuencia de individuos con el desenlace, lo que genera problemas de singularidad de la matriz hessiana en el modelo. El Criterio de Información de Akaike (AIC) evalúa modelos estadísticos equilibrando el ajuste a los datos con la complejidad de los parámetros. Se prefieren los modelos con menor AIC, ya que evitan el sobreajuste sin perder rendimiento. *RP = Razón de prevalencia; ^†^ Valor de p = Nivel de significancia; ^‡^ IC = Intervalo de confianza; ^§^ p < 0,001 = Significancia estadística; ^||^ SM = Salario mínimo, vigente en Brasil en 2023, R$ 1.320,00

En la Tabla 2 , el modelo 2 presentó el peor desempeño (AICc = 1951,693), lo que sugiere que la adición de las variables demográficas empeoró el ajuste, probablemente porque no contribuyeron significativamente a explicar el desenlace. A su vez, el modelo 3 presentó el menor AICc (1934,412), indicando el mejor equilibrio entre ajuste y complejidad representada por el número de variables incluidas.

El análisis multivariado jerarquizado de características asociadas a la probable dependencia de alcohol (AUDIT ≥ 20 puntos) evidenció que este desenlace fue más prevalente entre usuarios con menor nivel educativo (RP = 13,70; IC95% 1,56 – 117,63; p = 0,017) y que declararon educación superior incompleta (RP = 4,10; IC95% 1,09 – 15,42; p = 0,037) en comparación con aquellos con educación superior completa. También fue más prevalente entre usuarios con depresión en comparación con quienes negaron esta patología (RP = 2,72; IC95% 1,37 – 5,41; p = 0,005), conforme a la Tabla 3 .

**Tabla 3-:** Análisis multivariado jerarquizado de características asociadas a la probable dependencia de alcohol en usuarios de la atención primaria de salud (n = 40). São Paulo, SP, Brasil, 2023

	**Análisis jerarquizado**								
	**Modelo 1**			**Modelo 2**				**Modelo 3**	
**Nivel 1**	RP*	Valor de p ^†^	IC ^‡^ 95%	RP*	Valor de p ^†^	IC ^‡^ 95%	RP*	Valor de p ^†^	IC ^‡^ 95%
Sexo	p =0,174 ^§^			p =0,153 ^§^			p =0,106 ^§^		
Masculino	1,58	0,174	0,82; 3,05	1,59	0,153	0,84; 3,01	1,75	0,106	0,89; 3,45
Femenino	1			1			1		
Edad	**p =0,030**			**p =0,006**			p =0,269		
Años continuos	0,98	0,030	0,96; 1,00	0,97	0,006	0,96; 0,99	0,99	0,269	0,97; 1,01
Raza/color	p =0,774								
No declaró	1,89	0,534	0,25; 13,94						
Negra	0,94	0,904	0,33; 2,63						
Parda	1,34	0,416	0,66; 2,73						
Oriental	1,44	0,720	0,19; 10,63						
Indígena	3,51	0,203	0,51; 24,34						
Blanca	1								
**Nivel 2**									
Estado civil				p =0,208					
Viudo				2,58	0,224	0,56; 11,89			
Soltero				1,43	0,434	0,58; 3,52			
En unión libre				1,33	0,587	0,48; 3,68			
Divorciado/Separado				3,31	**0,021**	1,20; 9,13			
Casado				1					
Escolaridad				p =0,121			p =0,191		
Analfabeto				15,19	**0,015**	1,70; 135,98	13,70	**0,017**	1,60; 117,63
Educación primaria completa				3,25	0,142	0,67; 15,66	3,07	0,171	0,62; 15,29
Educación primaria incompleta				4,33	**0,025**	1,21; 15,54	3,88	0,051	0,99; 15,14
Educación secundaria completa				2,33	0,158	0,72; 7,55	2,21	0,214	0,63; 7,72
Educación secundaria incompleta				1,64	0,574	0,29; 9,24	1,42	0,694	0,24; 8,29
Educación Superior incompleta				4,67	**0,020**	1,28; 17,11	4,10	**0,037**	1,09; 15,42
Posgrado completo				1,25	0,854	0,12; 13,48	1,26	0,842	0,13; 12,21
Educación Superior completa				1			1		
Ingreso familiar				p =0,390					
De 1 a 5 SM ^||^				0,91	0,846	0,37; 2,24			
De 5 a 10 SM ^||^				2,09	0,234	0,62; 7,06			
≥ 10 SM ^||^				1,10	0,933	0,12; 9,70			
No sabe informar				0,49	0,317	0,12; 1,99			
< 1 SM ^||^				1					
**Nivel 3**									
Realiza actividad física							p =0,501		
No							1,26	0,501	0,64; 2,48
Sí							1		
Hipertensión arterial							p =0,400		
No							1,50	0,400	0,58; 3,82
Sí							1		
Diabetes							p =0,119		
No							3,11	0,119	0,75; 12,97
Sí							1		
Colesterol							p =0,961		
No							0,98	0,961	0,43; 2,23
Sí							1		
Problemas gástricos							p =0,851		
No							0,94	0,851	0,47; 1,86
Sí							1		
Depresión							**p =0,005**		
Sí							2,72	0,005	1,37; 5,41
No							1		
Criterio de Información de Akaike corregido	399,086			406,284			397,777		

Nota: Modelo 1 (ajustado para todas las variables del Nivel 1); Modelo 2 (ajustado para las variables con p < 0,200 en la prueba de efecto global del nivel anterior y para todas las variables del Nivel 2); Modelo 3 (ajustado para las variables con p < 0,200 en la prueba de efecto global de los niveles anteriores y para todas las variables del Nivel 3). La categoría “analfabeto” fue excluida del análisis debido a la baja frecuencia de individuos con el desenlace, lo que genera problemas de singularidad de la matriz hessiana en el modelo. El Criterio de Información de Akaike (AIC) evalúa modelos estadísticos equilibrando el ajuste a los datos con la complejidad de los parámetros. Se prefieren los modelos con menor AIC, ya que evitan el sobreajuste sin perder rendimiento. *RP = Razón de prevalencia; ^†^ Valor de p = Nivel de significancia; ^‡^ IC = Intervalo de confianza; ^§^ p < 0,001 = Significancia estadística; ^||^ SM = Salario mínimo, vigente en Brasil en 2023, R$ 1.320,00

En la Tabla 3 , el modelo 2 presentó el peor desempeño (AICc = 406,284), lo que sugiere que la adición de las variables demográficas empeoró el ajuste, probablemente porque no contribuyeron significativamente a explicar el desenlace. A su vez, el modelo 3 presentó el menor AICc (397,77), indicando el mejor equilibrio entre ajuste y complejidad representada por el número de variables incluidas.

## Discusión

El presente estudio tuvo como objetivo identificar la prevalencia de los patrones de consumo de alcohol entre usuarios de la APS y la asociación entre los patrones de consumo de riesgo, nocivo y probable dependencia con características sociodemográficas, clínicas y conductuales. Los resultados sobre la prevalencia de consumo de riesgo/nocivo en el presente estudio fueron del 17,1%, difiriendo de un relevamiento en el municipio de São Paulo que identificó una prevalencia del 7,7% en la población general ^(15 )^ .

En el desglose por sexo, las mujeres en el presente estudio presentaron una prevalencia de consumo de riesgo/nocivo del 9,9%, frente al 3,7% del relevamiento municipal; mientras que en la población masculina fue del 7,2% y 12,4% respectivamente. Cabe destacar que el 70% de la muestra estaba compuesta por mujeres, lo que corrobora un estudio previo ^(16 )^ . En este contexto, esto puede reflejar una mayor percepción sobre el propio consumo, derivada del seguimiento continuo en la UBS y de una mayor conciencia sobre su proceso de salud-enfermedad.

A pesar de ello, en el análisis multivariado el sexo masculino fue asociado al patrón de consumo de riesgo/nocivo. Se entiende que este patrón está estrechamente ligado a factores culturales y a la atribución de roles sociales distintos. Mientras que el consumo de bebidas alcohólicas por parte de los hombres puede asociarse a la expresión de masculinidad y sociabilidad, las mujeres a menudo enfrentan normas sociales más restrictivas, que desalientan dicho comportamiento ^(17 )^ .

Los hallazgos presentan evidencias claras de consumo de riesgo y nocivo entre jóvenes, compatibles con una investigación previa que destaca una preocupante asociación entre el consumo precoz de bebidas alcohólicas y la adopción de otras conductas de riesgo ^(18 )^ . Se entiende que esta alta prevalencia de uso de alcohol entre jóvenes se asocia con la fácil accesibilidad a la sustancia, sumada a influencias sociales y culturales, lo que contribuye a la banalización de su consumo ^(19-20 )^ .

El consumo de riesgo/nocivo también se asoció a personas que se autodeclararon como negras, lo que revela la intersección entre racismo estructural y salud mental, en la cual los individuos son diagnosticados a partir del color de su piel, además de enfrentar mayores dificultades de acceso a los servicios de salud ^(21-23 )^ . Un estudio realizado con veteranos identificó que los hombres negros tenían entre un 23% y un 109% más de probabilidades de recibir un diagnóstico de trastorno por uso de alcohol en comparación con los hombres blancos, evidenciando el sesgo diagnóstico al que las personas negras están sometidas ^(21 )^ . Además, estudios destacan el menor acceso a los cuidados de salud de la población negra en comparación con la población blanca, enfatizando las consecuencias negativas en los resultados de salud de dicha población ^(22-23 )^ .

Se entiende que las desigualdades son perpetuadas por barreras institucionales y experiencias de discriminación en los servicios de salud, donde determinados grupos sociales son sistemáticamente excluidos y marginados en las políticas de atención en alcohol y otras drogas, reproduciendo violencias históricas e institucionales ^(24-26 )^ . Este escenario demanda una reformulación urgente de las políticas públicas de salud mental y de la formación profesional, incorporando una perspectiva antirracista y culturalmente sensible que reconozca y enfrente activamente los marcadores sociales de las desigualdades. Tal enfoque es fundamental para desarrollar estrategias de atención que promuevan la equidad y rompan con los ciclos de exclusión y estigmatización.

A pesar de haberse encontrado en el estudio una asociación del consumo de riesgo/nocivo de alcohol con personas de mayores ingresos, esta se revela como compleja. Estudios señalan que individuos de bajos ingresos tienden a consumir alcohol de forma más frecuente e intensa, como respuesta a adversidades de tipo socioeconómico y psicológico ^(24,27 )^ ; en contrapartida, personas de altos ingresos presentan un consumo elevado, impulsado por factores culturales y sociales, como eventos y demostración de estatus ^(28-29 )^ . Además, el acceso a cuidados y apoyo en salud es mayor entre los individuos de mayores ingresos, lo que puede agravar los desenlaces para los económicamente vulnerables, quienes enfrentan barreras significativas para buscar tratamiento ^(30 )^ .

Cabe destacar además que la pandemia intensificó estos desafíos. Estudios señalan implicaciones importantes para la accesibilidad al consumo debido a impactos financieros, además de políticas públicas de control del consumo ^(31 )^ . Y aunque no se haya observado una asociación significativa entre el consumo perjudicial de alcohol y los impactos directos de la pandemia en la muestra analizada, es importante señalar que la literatura indica un aumento generalizado de conductas de riesgo para la salud durante el período de aislamiento social ^(32 )^ .

Este escenario sugiere que el contexto de crisis sanitaria pudo haber exacerbado factores de riesgo para el uso problemático de alcohol en otras poblaciones, por lo que resulta imprescindible implementar medidas preventivas en la APS, considerando las múltiples dimensiones que influyen en los patrones de consumo y su relación con los factores socioeconómicos de la población.

Las comorbilidades más autodeclaradas fueron hipertensión, diabetes mellitus, gastritis y colesterol. A pesar de la ausencia de significancia estadística, los datos corroboran con la literatura, que señala una alta prevalencia de enfermedades crónicas preexistentes entre individuos que presentan consumo perjudicial de alcohol en la APS, lo que resulta en el agravamiento de dichas condiciones ^(33-34 )^ . La falta de un abordaje sistemático del consumo de alcohol contribuye al subdiagnóstico y a la ausencia de prevención y tratamiento precoz de los trastornos relacionados con el alcohol ^(35 )^ , lo que resalta la necesidad de una mayor atención al cribado e intervenciones en la APS.

Es importante destacar que, por tratarse de un estudio transversal, no es posible establecer relaciones causales o temporales entre el consumo de alcohol y las comorbilidades identificadas. La complejidad de estas asociaciones puede ir más allá de los límites de un estudio transversal, dado que factores socioculturales, económicos y conductuales –no incluidos en el modelo estadístico– pueden desempeñar un papel importante en dichas asociaciones, por lo que se recomiendan estudios longitudinales que incorporen una gama más amplia de variables para esclarecer tales cuestiones, como sugerencia para investigaciones futuras.

Los resultados de este estudio señalaron una asociación entre el nivel educativo, la depresión autodeclarada y la probable dependencia de alcohol, en consonancia con estudios previos ^(20,36 )^ . Se entiende que la educación desempeña un papel crucial en el desarrollo de habilidades de afrontamiento y en la formación de redes de apoyo social, factores que contribuyen a la prevención de la dependencia; por otro lado, su ausencia puede limitar el acceso a estas herramientas de protección, favoreciendo comportamientos de riesgo ^(37-38 )^ . Considerando que el alcohol es frecuentemente utilizado como una estrategia para aliviar los síntomas del sufrimiento mental, como la ansiedad y la tristeza, se destaca la necesidad de abordajes integrados que consideren tanto los aspectos psicológicos como los sociales para una mejor derivación de los casos ^(22-34 )^ .

Tal como lo evidencian los resultados de este estudio, los problemas relacionados con el consumo de alcohol están influenciados tanto por factores individuales como por determinantes sociales. Por lo tanto, la APS, con su carácter integral y territorializado, se presenta como un espacio estratégico para acciones de promoción de la salud, prevención de agravios y reducción de daños. Sin embargo, para que estas estrategias sean efectivas, es imprescindible superar las barreras organizacionales existentes, como la alta demanda de atención y la sobrecarga de los profesionales, además de invertir en la capacitación de los equipos para un abordaje libre de estigmas y centrado en la acogida.

Este estudio debe interpretarse en el contexto de sus limitaciones, entre ellas que todos los resultados se basan en autoinforme, lo que puede llevar a la omisión de información o a la subestimación de la frecuencia y cantidad de alcohol consumido, así como a limitaciones en el acceso a servicios especializados, debido a posibles sesgos de deseabilidad social. Se implementaron estrategias para la estandarización de la recolección de datos y la alineación de los investigadores con el fin de minimizar dicho sesgo.

Además, el diseño transversal también limita la inferencia causal, ya que no es posible determinar la temporalidad entre la exposición (consumo de alcohol) y los desenlaces (como comorbilidades y trastornos mentales). La recolección de datos durante la pandemia puede haber introducido sesgo de memoria, además de la falta de datos comparativos previos a la pandemia, lo que limita la capacidad de evaluar cambios específicos en ese período. Asimismo, el hecho de que la muestra no sea representativa del municipio ni de otras regiones imposibilita la generalización de los datos.

Asimismo, variables como religión/religiosidad, apoyo social, otros factores protectores conocidos y la exclusión de personas sordas no fueron consideradas, lo cual puede representar una limitación adicional. No obstante, los hallazgos de este estudio pueden servir de base para futuras investigaciones sobre la prevalencia de los patrones de consumo de alcohol entre los usuarios de los servicios de APS, que consideren los aspectos señalados.

## Conclusión

Se concluye que el estudio, al buscar identificar la prevalencia de los patrones de consumo de alcohol entre usuarios de la APS en la ciudad de São Paulo, señaló que el consumo perjudicial de alcohol está asociado principalmente a usuarios del sexo masculino, más jóvenes, de raza/color negra y con ingresos iguales o superiores a 10 salarios mínimos. La búsqueda de atención en la APS ocurre, en su mayoría, por otros problemas de salud, lo que refuerza la importancia de este nivel de atención como un escenario estratégico para la identificación precoz del consumo perjudicial de alcohol y la implementación de medidas preventivas.

## Data Availability

Todos los datos generados o analizados durante este estudio están incluidos en este artículo publicado.
